# Commercial-Scale Modification of NdFeB Magnets under Laser-Assisted Conditions

**DOI:** 10.3390/nano14050431

**Published:** 2024-02-27

**Authors:** Natalia Radwan-Pragłowska, Julia Radwan-Pragłowska, Karol Łysiak, Tomasz Galek, Łukasz Janus, Dariusz Bogdał

**Affiliations:** 1 Faculty of Electrical and Computer Engineering, Cracow University of Technology, Warszawska 24 St., 31-155 Cracow, Poland; 2 Faculty of Chemical Engineering and Technology, Cracow University of Technology, Warszawska 24 St., 31-155 Cracow, Poland; 3 Faculty of Mechanics and Technology, Rzeszow University of Technology, Kwiatkowskiego 4 St., 37-450 Stalowa Wola, Poland

**Keywords:** laser surface modification, permanent magnets (PM), nanostructured coatings, magnets’ surface protection

## Abstract

Rare Earth elements (REE) such as NdFeB are commonly used to produce permanent magnets. Thanks to their superior properties, these materials are highly desirable for green energy applications such as wind power generators or electric cars. Currently, REEs are critical for the ongoing development of eco-friendly solutions in different industrial branches. The emerging issue of REE depletion has led to a need for new methods to enable the life cycle elongation, resistance to wear, and external factors improvement of NdFeB magnets. This can be achieved by advanced, nanostructured coating formation of magnet surfaces to increase their functionality and protect from humidity, pressure, temperature, and other factors. The aim of the following research was to develop a new, scalable strategy for the modification of NdFeB magnets using laser-assisted technique, also known as Laser cladding. For this purpose, four different micropowders were used to modify commercial NdFeB samples. The products were investigated for their morphology, structure, chemical composition, and crystallography. Moreover, magnetic flux density was evaluated. Our results showed that laser cladding constitutes a promising strategy for REE-based permanent magnets modification and regeneration and may help to improve durability and resistance of NdFeB components.

## 1. Introduction

Magnets, i.e., bodies that produce a permanent magnetic field, are widely used in both everyday appliances (household appliances) as well as in larger electric machines (including those that work with Renewable Energy Sources (RES)), as well as hybrid and electric cars [1,2].

The following materials are most commonly used: neodymium magnets (NdFeB), AlNiCo magnets, Samarium-Cobalt magnets, and ferrite magnets (Barium and Strontium). The strongest among these are neodymium magnets. Magnetic materials based on rare earth elements are crucial for green energy generation as they are used in production of wind turbines and electric vehicles as well as in actuators and sensors. NdFeB magnets are also used for automation. Their utility is diverse as they can be tailored in terms of both shape and geometry. Nevertheless, obtaining materials for manufacturing magnets from rare earth elements is not only a demanding process but is also costly and time consuming [3,4,5,6,7].

Though they are characterized by superior magnetic properties such as large maximum energy product, high remanence, and coercivity, NdFeB magnets require the use of special coatings due to their susceptibility to corrosion; this is caused by their porous and loose nature, which make them prone to degradation under humidity or high temperature. Therefore, before they find commercial applications, they must be subjected to modification to increase their resistance to external environmental factors. The search for solutions to increase this level of protection using different magnet coatings has been performed in many studies [3,4,8,9,10]. Among the most commonly used coatings are Ni-Cu-Ni, Zn, Rubber, Au, PTFE, Cr, and Ti Nitride [5,6,7,8,9,10]. Coating manufacturing methods include the best-known example, based on electrochemical processes [8], or otherwise surface polishing by industrial robots and cold-sprayed coatings used for smaller scale [7,11,12,13,14,15]. In this article, the authors propose the use of laser-assisted treatment to modify the magnets’ surface. 

There is an emerging issue of rare earth metal depletion; this constitutes a significant problem for manufactures whose machines rely on the use of NdFeB magnets. Therefore, development of a novel coating strategy that would increase the product lifetime is a of the greatest importance [16,17,18,19,20]. A very promising method relies on the use of laser-assisted techniques since they enable fast, efficient, repeatable, and scalable surface modification thanks to high energy density, favorable coherence, and direction. This method can be easily implemented by various manufacturers, who, as a result, would be capable of independent regeneration of damaged magnets or their coatings using already owned equipment such as laser sources or robotic stations. Laser cladding is a modern technique that has been already implemented in different industrial branches, especially automotive, aerospace, or petrochemistry, where the high precision of detailed production and finish is strongly desired. It enables preparation of complexed parts characterized by superior properties such as increased resistance to external factors like temperature, pressure, or humidity [21,22,23,24]. This method combines not only laser technology but also automated control and computer-assisted manufacturing. However, despite the high degree of automatization, the skills of the operator have a significant impact on the success of the process since laser-cladding is associated with high energy generation, which must be managed properly. It provides the possibility to obtain semi-products from raw materials such as various alloys which have great mechanical properties and immune to wear. This modification technique enables fast powder melting under laser beam irradiation followed by solidification at the surface of the substrate, resulting in formation of a uniform layer with the desired properties. Laser cladding produces a tough and fine-grained coating due to the possibility of large temperature gradients, energy focus, and heat dispersion. Compared to other techniques, it is a simple, fast, and efficient method that enables powder saving and recycling, as well as precise surface modification. Moreover, laser cladding gives the possibility to obtain desired nanostructures while maintaining small dilution zone and heat affected area [25,26,27,28,29,30,31,32,33].

One of the most emerging issues concerning laser cladding is the development of novel powder formulas that would provide satisfactory mixing with the substrate. The right choice of size, morphology, and chemical composition is crucial to obtain a homogenous, durable layer. During the process, powder undergoes melting followed by crystallization. As a consequence of laser beam interaction with the modifying agent, the particles first adsorb the powder, leading to temperature increase, then the energy is weakened. As such, it is crucial to adjust the parameters properly, according to specific powder characteristics. Its melting point should be lower than the workpiece to prevent modified material degradation and shape destruction. Furthermore, laser beam/powder/substrate interactions are also different depending on whether particles are ceramics or metal. Metallurgical miscibility determines whether the powder will melt after laser beam irradiation and act as a binder or whether it will harden the surface, which is undesirable effect. Appropriate in-melt of the particle increases physical durability and the mechanical efficiency of the material. This method distinguishes two different approaches depending on the powder type and desired modification result. The first is considered to be a one-step method since the powder is fed in a synchronous manner and no surface pretreatment is needed. The second involves two steps. First, the workpiece is covered by the powder particles without forming chemical bonds, and then the laser beam scans it in a rapid manner. Laser cladding is superior to other traditional surface modification methods since it can be used for different workpieces and substrates coating. In addition, versatile powders can be applied as modifying agents. Thus, it constitutes a versatile tool for various material systems. Moreover, it provides both rapid heating and cooling, and so layers can be significantly reinforced and refined. Thanks to the laser beam’s focus, efficient energy management is possible and minimal waste is generated. The process by itself can be considered economical as it does not require high dilution and the heat-affected zone of the raw material is considerably small; as such, no workpiece degradation occurs, thus reducing production losses. Therefore, it can be called an environment-friendly method. 

The aim of this article was to develop a novel, large-scale method for surface modification of NdFeB magnets to increase their stability in regard to external factors and to improve their life cycle and wear resistance via the formation of nanostructured coatings.

## 2. Material and Methods

### 2.1. Materials

Spongy titanium (titanium powder) was obtained from Ireneusz Katarzyński Selkat. Zircon dioxide powder was purchased from Alchem Sp. z o. o. NiBSi/ZrO_2_ and Nd/ZrO_2_ (9:1 ratio) powder mixes were obtained using a ball mill. Raw, cuboid Nd-Fe-B magnets (40 × 18 × 10 mm) were purchased from Enes Magnesy Paweł Zientek Sp. k. The magnetic parameters of the N38 PM magnets are as following: coercivity H_c_B: min. 899 [kA/m]; H_c_J: min. 955 [kA/m], remanence B_r_: 1.21–1.25 [T], and energy density (BH)_max_: 286–302 [kJ/m^3^].

### 2.2. Coating Preparation

**Figure 1:**
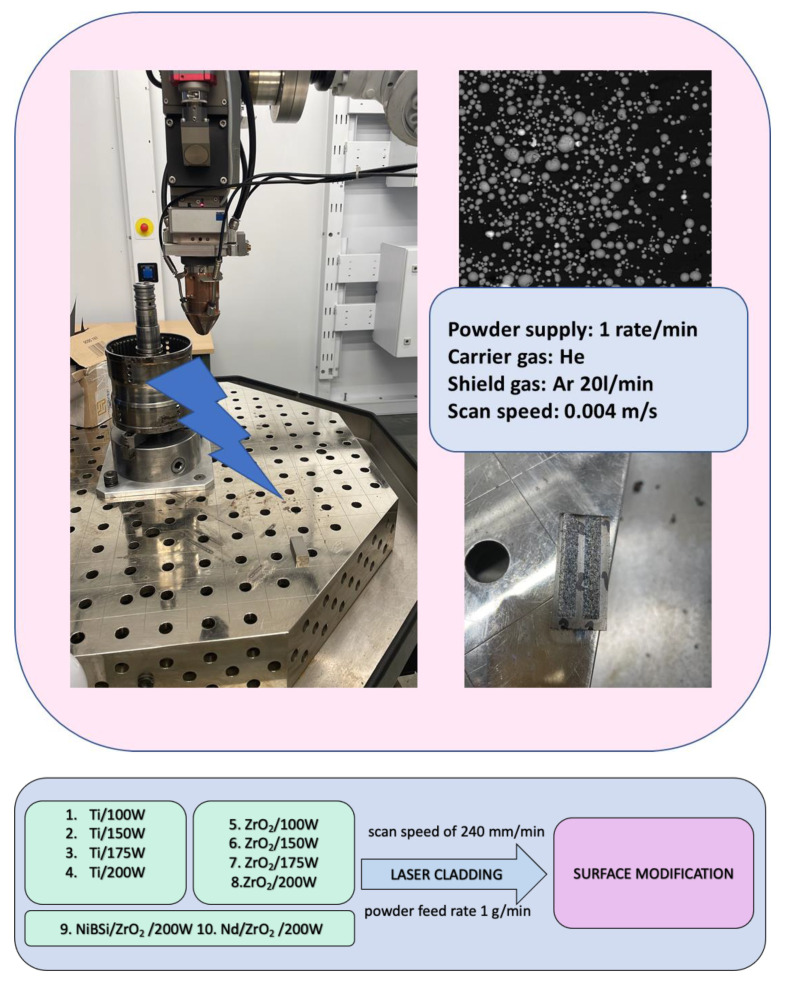
Process parameters.

The samples were modified using diode pumped TRU LASER ROBOT 5020 with parameters robot arm capacity: 30 kg, type of source-disk laser, beam quality of 8 mm*mrad with light wavelength of 1030 nm and output power on the workpiece: min. 4000 W. The workstation for the experiments also involved a KUKA KR30 HA robot. A three-stream head was used integrated with a metal powder feeder. To perform the surface modification of the magnet and to provide uniform cladding, pre-treatment was carried out to clean the surface of contaminants. First, it was polished with sandpaper and then treated with isopropanol to remove dirt, dust, and grease. Coatings were obtained using four types of powders, namely spongy Ti (Sample 1), ZrO_2_ stabilized with Y (Sample 2), and self-made NiBSi/ZrO_2_ (Sample 3) and Nd/ZrO_2_ (Sample 4) powder mixes. Powder feeding was one rate per minute. ZrO_2_ stabilized with Y feeding was 2.2 g/min, spongy titanium 1 g/min, NiBSi/ZrO_2_ 3.4 g/min, and Nd/ZrO_2_ 2.4 g/min. To provide effective melting carrier gas, He was used to feed the powder. To protect the substrate from oxidation and laser beam energy dispersion, a shield gas was used (Ar). Four different power values were applied, 100, 150, 175, and 200 W, for the samples modified with Ti and ZrO_2_, while for NiBSi/ZrO_2_ and Nd/ZrO_2_, 200 W was used (Figure 1). Scan speed during modification was 240 mm per min.

### 2.3. Surface Properties Study

SEM analysis and surface elemental composition were carried out using a Tescan HR-SEM/EDS scanning electron microscope. For image collection, the BSE mode was used. Roughness of the samples was determined using Fiji J Image software version ImageJ-win64. XRD analysis was carried out using a Bruker apparatus. XPS analysis was performed using XPS K-Alpha photoelectron spectrometer from Thermo Fisher Scientific (Warsaw, Poland) to analyze coating thickness and in-melt efficiency. 

### 2.4. Magnetic Flux Density Tests

To verify the laboratory tests, field calculations were performed using FEM (Finite Element Method). The authors used the ANSYS Maxwell software 2020 R2 to prepare the magnetostatic analysis of neodymium PM magnets. The simulation temperature was 20 °C and magnet’s dimensions were 20 × 10 × 5 mm.

**Figure 2:**
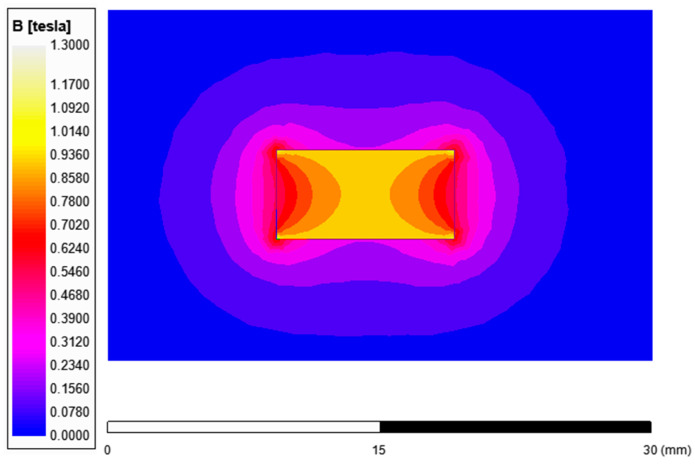
Permanent magnets’ flux density distribution (cross-section) for magnet type N38, 20 × 10 × 5 mm.

Figure 2 shows the magnetic flux distribution for the PM magnet of type N38. The performed magnetostatic analysis presents the magnetic field distribution not only for the magnets but also for the surroundings, which extends the results obtained from laboratory tests.

The magnetic flux density concentrates in the middle of the magnet and decreases in both directions towards the edges, reaching 540 mT for magnet 20 × 10 × 5 mm. 

According to the field analysis, measurements of magnetic flux density *B* [T] were taken. The magnetic flux density was measured using a Teslameter TENMARS TM 197 positioned 5 mm from the magnet’s surface, as per the Teslameter’s documentation. Two cases were compared: a raw magnet (without any coating) and a magnet with commercial coating (Ni-Cu-Ni), both 20 × 10 × 5 mm. Both magnets had the same dimensions and product datasheet. However, it can be seen that the commercial coating, which increases resistance to external conditions, lowered the initial induction value at room temperature (the difference is close to 7%). 

Additionally, when analyzing the results, we noticed the magnetic flux density value, obtained from laboratory tests at a distance of 5 mm from the magnet’s surface (size 20 × 10 × 5 mm, no coating, room temperature), was 116 mT. According to the field analysis value, the flux density at this point was 120 mT. The magnetic flux density value, measured for the samples after the coating process, ranged from 10 mT to 80 mT (depending on the laser power, from 200 W to 100 W). As a result, the samples had to be re-magnetized. 

## 3. Results and Discussion

### 3.1. Morphology Study

**Figure 3:**
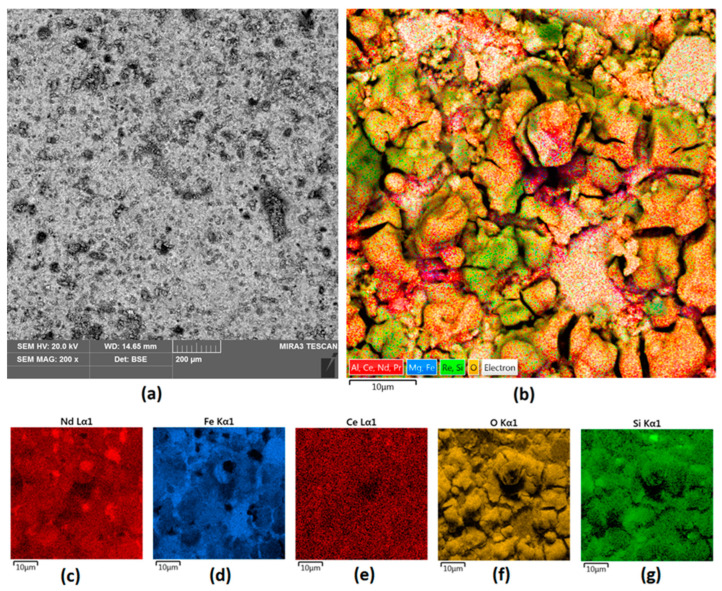
(**a**) raw neodymium magnet surface 200× magnification; (**b**) elemental analysis of the unmodified Nd magnet surface—mapping; (**c**–**g**) elemental analysis of the Nd magnet surface—mapping (**c**) Nd; (**d**) Fe; (**e**) Ce; (**f**) O; (**g**) Si.

Commercially, neodymium magnets are electrochemically coated with Ni to provide resistance to environmental factors and to increase their durability. However, such coatings have some pitfalls associated with susceptibility to degradation in highly demanding environments, especially when magnets are used as components in wind turbines situated on the coast or in the sea. As shown in Figure 3a, the surface of uncoated NdFeB magnets is highly heterogeneous and porous. In addition, some superficial defects are present, as well as some inclusions. Figure 3b, together with Figure 3c–g, shows the elemental composition of the unmodified material. It can be noted that, other than Nd and Fe, some other rare earth elements are present, including Ce, Pr, or Re, which shows that commercial magnets contain some contaminants. Oxygen is also present, which confirms superficial oxidation of the reactive elements such as Nd or Fe. Presented results show, without a doubt, that unmodified NdFeB magnets are susceptible to chemical degradation and undergo reactions with environment components.

It can be seen that NdFeB magnets without protective coating exhibit heterogeneous morphology and have a porous structure, which may be a consequence of superficial oxidation as well as micro-destruction due to the brittle nature of the material.

As shown in Figure 3, bare magnets need superficial coverage to prevent them from environmental impact and mechanical factors. Therefore, an attempt was made to verify whether it is possible to provide superior protection outcomes when compared to traditional electrochemical coating protection. Laser cladding is considered to be one of the most promising methods for fast and efficient surface modification as it enables the precise delivery of power to target area. 

For small experiments, two-step modification seems to be the most reasonable. In this method, the magnet is covered with the powder in the form of, e.g., a paste prepared by mixing its particles with inert, non-reacting solvent (water, isopropanol etc.) that evaporates during laser beam scanning due to heat generation. However, pre-placing the pathway is more time consuming and less efficient due to significant powder loss compared to the one-step method. This latter approach enables simultaneous powder feeding and cladding, making it easily scalable and possible to implement at industrial scales. The process starts with laser beam generation, which, due to its energy being absorbed by the powder particles, enables its rapid melting, followed by solidification and coating formation. When both parameters and powder physiochemical properties are well adjusted, a uniform and durable layer is formed. NdFeB magnets are known for their brittle nature, which significantly impedes their modification. Therefore, the first attempts at laser cladding were carried out by applying low power in order to prevent cracking because of a too high energy delivery since the substrate is also exposed to laser beam treatment and its surface undergoes melting with the applied powder.

**Figure 4:**
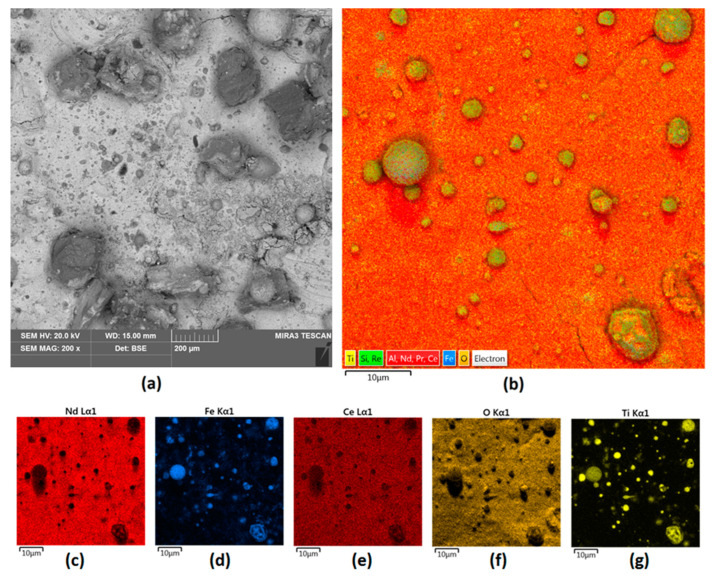
Sample 1 appearance (**a**) 200× magnification; (**b**) elemental mapping; (**c**–**g**) elemental analysis of the Nd magnet surface—mapping (**c**) Nd; (**d**) Fe; (**e**) Ce; (**f**) O; (**g**) Ti.

After the first attempts at cladding using spongy Titanium as a modifier in the synchronous powder feeding approach at powers of 100 W, 150 W, and 175 W, we performed tests with a power of 200 W. At 100 W, 150 W, and 175 W, some changes in surface morphology and the formation of nanostructured regions occurred, but the power was too low to provide in-melt. The Ti content, according to EDS analysis (Supplementary Material; the EDS data are given in weight percent), was below 1%, which proves that such parameters are insufficient. Increasing the power to 200 W resulted in better mixing of the Ti powder with magnet and its nanoscale dispersion. Nevertheless, higher in-melt was associated with phase separation, and some superficial granules are present (Figure 4). Although the Ti content in the chemical composition increased to 3.6% overall, it can be stated that pure Titanium powder cannot be considered as a good material for surface modification at low power despite the highly porous nature of its particles.

Another compound known for its superior properties in terms of durability enhancement is ZrO_2_. Tests were carried out using laser-assisted modification using metal oxide powder stabilized with yttrium. ZrO_2_ particles were characterized by their spherical shape and a diameter below 100 µm. 

After performing the test at 100 W, visible changes in sample morphology were noted. Embedded grains with a size of 10 µm homogenously covered the whole surface. The obtained coating was quite uniform and rough. No separate phases were distinguished. Elemental mapping showed that Zr distribution was even. The amount of melted powder at 150 W was higher when compared to spongy titanium; zirconium content was 0.6% according to the EDS analysis (the EDS data are given in weight percent). Further increasing the applied power (150 W) in this case significantly improved the amount of powder that was in-melted. The relatively thick coating was formed but the coverage was not uniform, and it was noticed that part of it fell off the surface, despite the magnets receiving pre-treatment, and separated phases could be distinguished. This phenomenon can be explained by the energy application being too low or the exposition being too short, meaning that the power output was enough to melt the powder but not to fully attach it to the magnet. Zr was present in the whole magnet but in different amounts. Nevertheless, the total content of this element increased to 12.2% according to EDS analysis.

Subsequently, the authors performed magnet modification with ZrO_2_ at a the power of 175 W. It was noticed that the morphology of the surface was different compared to the previous samples to which lower power was applied. A zircon oxide-formed mesh covered the magnet with only few particles left exposed, which suggests that the increased power was high enough to prevent power dispersion before mixing with the material surface. As a result, the nanostructured architecture was obtained. Nevertheless, separate phases were again visible, and the coverage was not fully homogeneous. At the same time, it was noticed that Zr content was above 40%, according to the EDS analysis, which showed that the power increase was followed by a greater amount of embedded powder.

**Figure 5:**
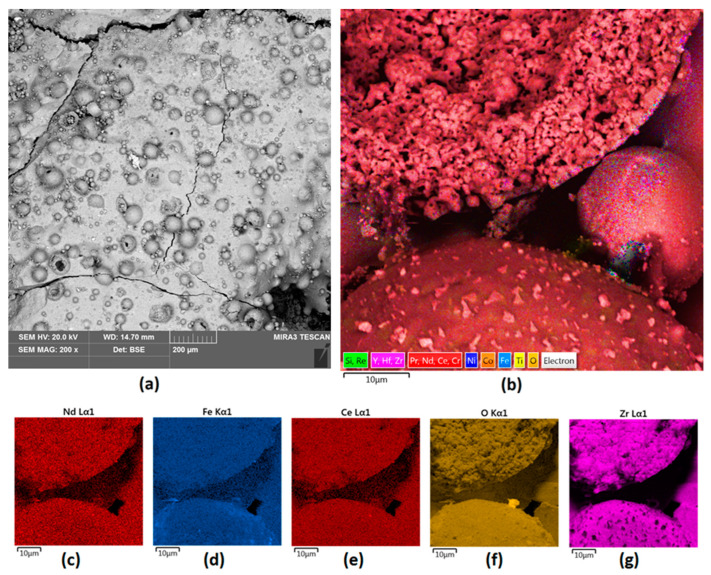
Sample 2 (**a**) 200× magnification; (**b**) elemental mapping; elemental analysis of Sample 2 surface—mapping (**c**) Nd; (**d**) Fe; (**e**) Ce; (**f**) O; (**g**) Zr.

Figure 5 shows the results of ZrO_2_ cladding when applying a power of 200 W. Such parameters ensure the full melting of the powder and its mixing, followed by permanent bonding with the surface. However, it can be seen that such high power also resulted in bubbles that looked like small crater formations; these can be assigned to entrapped gases. Some superficial defects, such as cracks, can be spotted, which formed because of the dense coating and internal stress. Figure 5 shows that ZrO_2_ fully dominated the magnet’s surface. Zr content is above 56%, proving that the spherical morphology of the powder as well as the oxide form of the metal provide suitable conditions for laser cladding. Importantly, nanoporous regions are visible, which are responsible for potential increased adhesiveness and surface area. 

Our experiments show that optimized parameters provide better sample coverage. However, applying Ti and ZrO_2_ powders does not enable preparation of the coating, which would be characterized by uniformity and in-melt, at the satisfactory level from the commercial point of view. 

**Figure 6:**
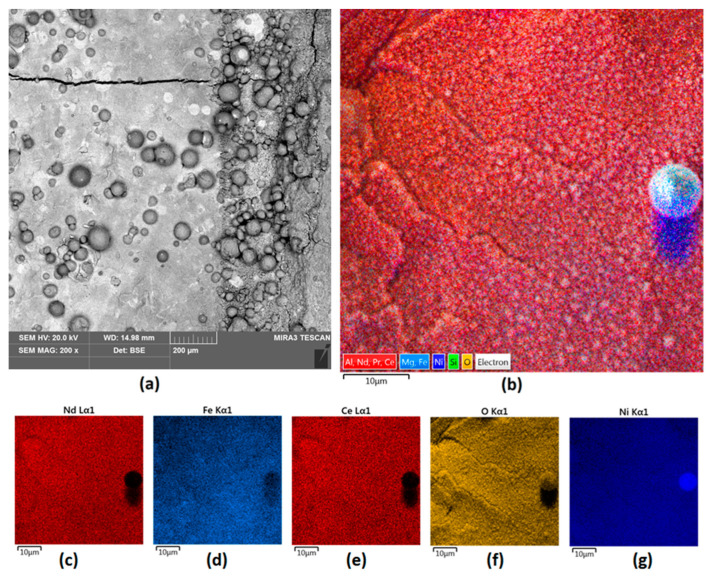
Sample 3 (**a**) surface 200× magnification; (**b**) elemental analysis of the Nd magnet surface—mapping; elemental analysis of Sample 3 surface—mapping (**c**) Nd; (**d**) Fe; (**e**) Ce; (**f**) O; (**g**) Ni.

As applying more than 200 W of power to NdFeB magnets results in cracking due to their brittle nature, another attempt at superficial modification was made using two other powders that have been successfully used for the laser cladding of commercial steel. Figure 6 presents the results of magnet modification conducted by applying a mix of NiBSi with ZrO_2_ (9:1) at a power of 200 W. It can be seen that preparation of the composite powder using raw materials of the similar granulation resulted in a fine, nanostructured uniform layer with a significantly greater surface homogeneity compared to previous samples. No separate phases can be distinguished. 

**Figure 7:**
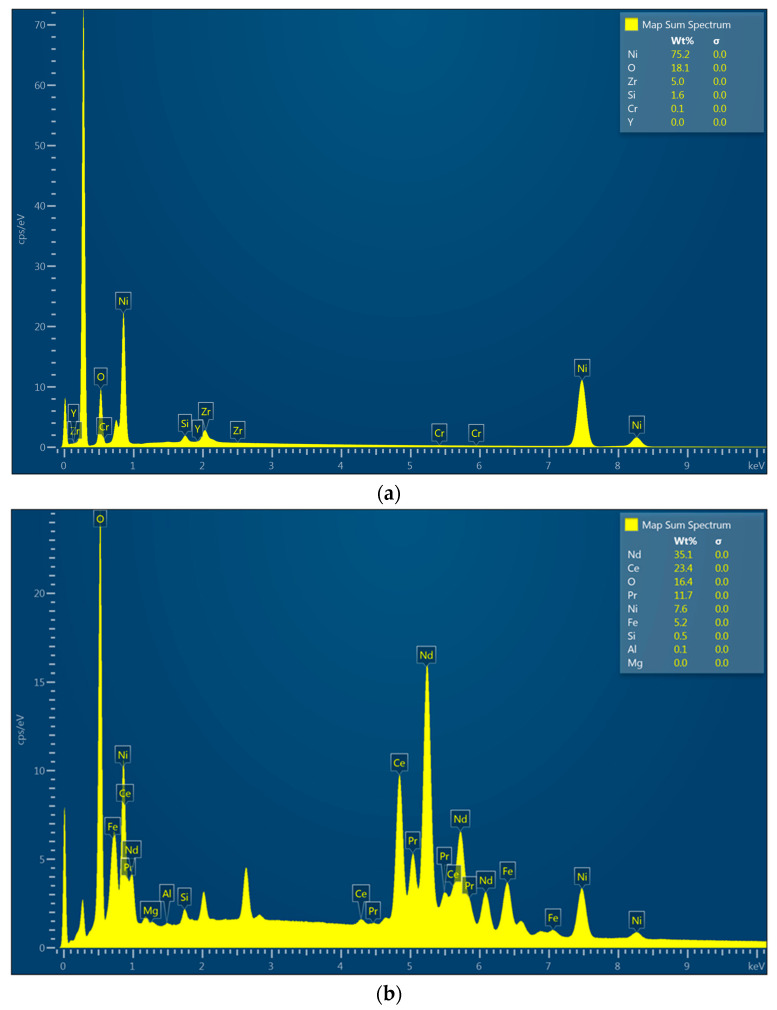
(**a**) EDS analysis of powder used for Sample 3 coating; (**b**) EDS analysis of Sample 3.

Figure 7a,b shows the results of the EDS analysis performed for the NiBSi/ZrO_2_ powder and for Sample 3. It can be seen that the pure powder spectrum confirms the presence of elements such as Ni, O, Zr, and Si in its composition. Comparing these two spectra, it is clear that successful mixing of the powder with the substrate has occurred and that in-melt was achieved. This is because the elemental composition of the sample surface after modification reveals the presence of elements present in both the unmodified magnet, namely Nd, Ce, Pr, and Fe, and those derived from the powder, especially Ni, which is present as a result of its high content in the modifying medium. In this case, the Ni content was almost 8%, and thus it can be assumed that the used powder constituted around 11% of the investigated surface.

**Figure 8:**
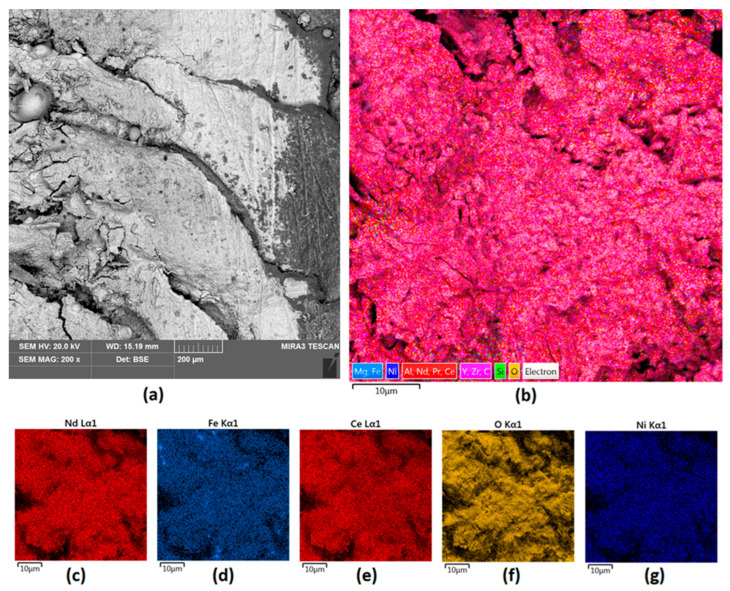
Sample 4 (**a**) surface 200× magnification; (**b**) elemental analysis of the Nd magnet surface—mapping; elemental analysis of Sample 4 surface—mapping (**c**) Nd; (**d**) Fe; (**e**) Ce; (**f**) O; (**g**) Ni.

The last attempt at modification was carried out using a mix of Nd and ZrO_2_ powders. As shown in Figure 8, 200 W of power was enough to provide full coverage of the surface. Nevertheless, it was correlated with the formation of cracks and hollows due to the problems of energy dispersion during the cladding process. The newly formed coating is heterogenous and highly porous (both micro and nanopores are present), which may negatively affect the future stability of the magnet in high demand environments and does not protect it from corrosion. 

**Figure 9:**
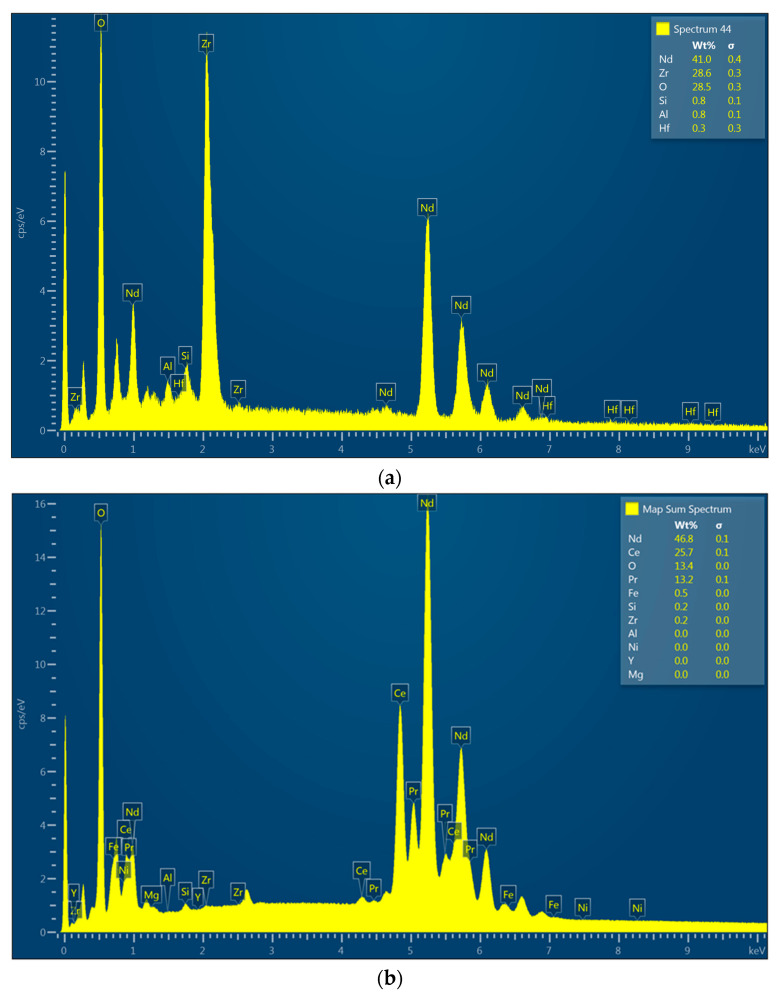
(**a**) EDS analysis of powder used for Sample 4 coating; (**b**) EDS analysis of Sample 4.

Figure 9a,b presents the EDS spectra obtained for the Nd/ZrO_2_ powder and for the final Sample 4. The powder’s spectrum shows the presence of Nd, Zr, and O. The same elements are also present in Figure 9b, which confirms the successful cladding and powder mixing with the bed as a consequence of laser beam treatment. This leads to particles melting since elements like Ce, Pr, and Fe are present and could be distinguished in the untreated magnets. In all cases, no peaks corresponding to B can be noticed; this is due to the physicochemical properties of this element, which make it difficult or impossible to visualize using the EDS technique.

SEM analysis shows that the best in-melt and coverage was obtained for Samples 3 and 4, and that the surfaces were characterized by the lowest heterogeneity and surface defects. Samples that were modified by applying 100 W and 150 W of power, respectively, exhibit rather low roughness; this is explained by the small amount of powder that merged with the magnet. 

Samples modified with higher powers are characterized by diverse surface structures due to the in-melt of the powders and their mixing with internal layers of the magnet. Taken together, it can be assumed that the most favorable surfaces were obtained during modifications with 200 W of ZrO_2_ and ZrO_2_-based powders.

Coating thickness and heat affecting zone size are other important parameters for commercial applications of superficially modified materials. As shown in Table 1, the coating thickness for the investigated samples was between 297 and 1351 µm. The thinnest was obtained for the sample clad using spongy titanium at 200 W and the thickest for NiBSi/ZrO_2_ powder treated at 200 W. Coating thickness decreased as the power increased; this can be explained by better in-melt and the resultant mixing with the substrate due to the higher energy dose. The thickest coating was obtained for NiBSi/ZrO_2_ at 200 W, which could be a consequence of both particle size and powder morphology as well as its density. 

**Figure 10:**
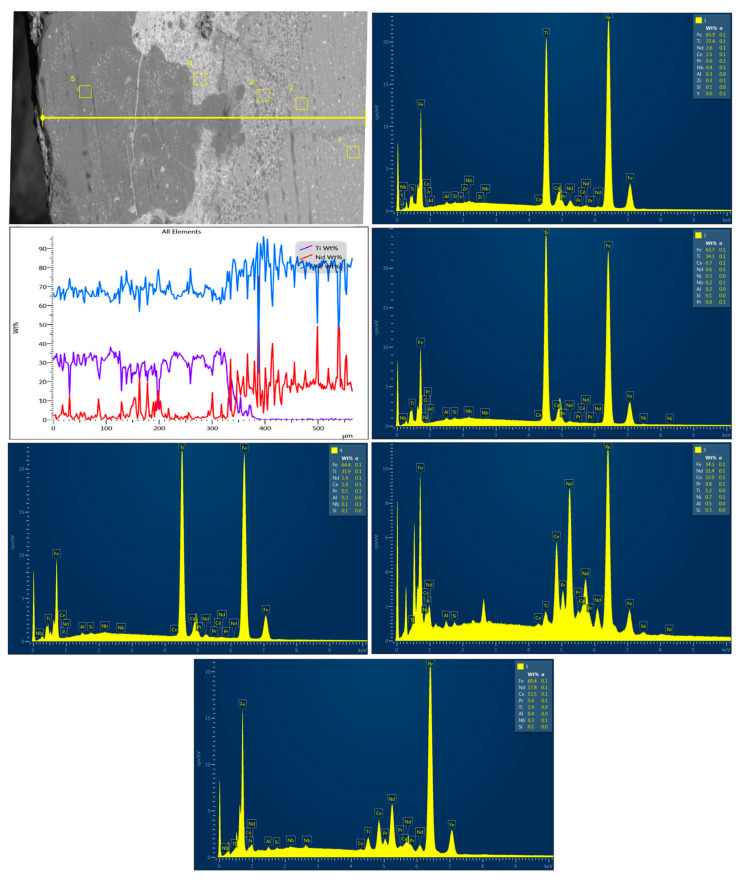
Ti/100 W cross-section with elemental composition of the cross-section and EDS analysis. Numbers 1–5 at the cross-section represent points of analysis and are correlated with EDS spectra.

Figure 10 reveals a cross-section of the Ti/100 W sample. It can be seen that the powder is well mixed with the substrate and that no heating zone can be distinguished, which is typical for ceramic or composite materials. It can be seen that pure titanium constitutes a layer of 300 µm. In addition, a hybrid, well-mixed layer can be distinguished, which is a characteristic phenomenon for laser-assisted powder cladding. Importantly, four different phases can be distinguished. The outer zones (no. 1 and 2) are composed of about 30% of titanium, which confirms mixing with substrate, and reveals a surprisingly high content of Fe. Interestingly, one of the deeper zones (no. 3) shows a lower content of Fe and an increased percentage of both neodymium and cerium; this can be explained by the differences in melting temperatures (approximately 2500 °C Fe and 1000 °C Nd and Ce, respectively). In addition, very small quantities of Ti can be distinguished. The next zone (no. 4) again reveals a high content of modifying powder, similar to zones 1 and 2. This suggests a sudden and intense mixing as a consequence of laser beam treatment associated with high power dose delivery. The last (5th) zone is composed of the basic NdFeB magnet elements, which proves that the in-melt range is about 380 µm. It also confirms that mixing with basic material is not uniform and heterogeneous, which suggests that pure titanium is a demanding material for cladding.

**Figure 11:**
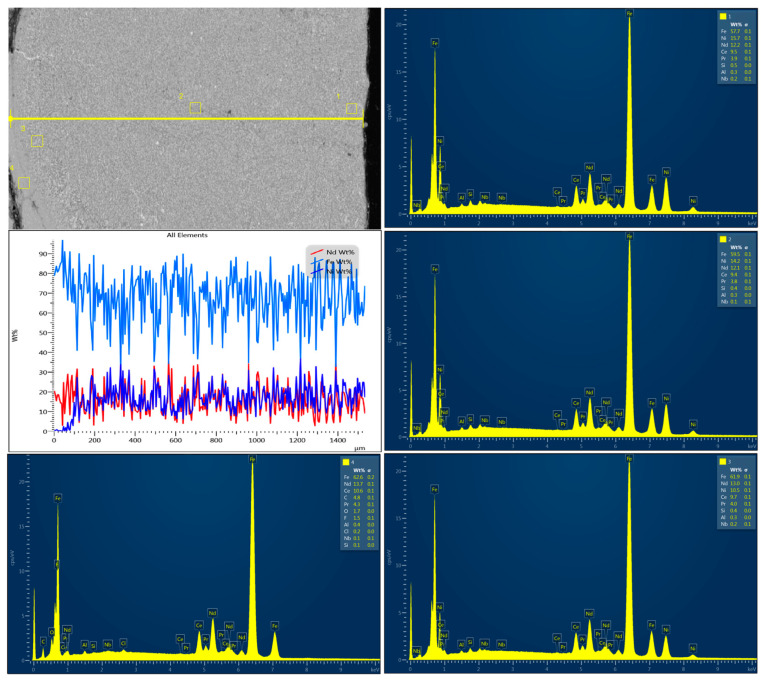
NiBSi/ZrO_2_ 200 W cross-section with elemental composition of the cross-section and EDS analysis. Numbers 1–4 at the cross-section represent points of analysis and are correlated with EDS spectra.

Figure 11 presents a cross-section of the NiBSi/ZrO_2_ 200 W sample. Unlike the Ti 100 W sample, the gradient change of Ni content can be noticed. The highest amount of the element mixed with the substrate is present at the inner zone. The deeper the beam penetration, the lower the content of the Ni, which shows homogenous dispersion and in-built modifying powder as shown in zone no. 3. The deepest zone (no. 4) is composed only of NdFeB magnet constituents. Therefore, coating thickness can be estimated as approximately 1350 µm. Thus, modification with NiBSi/ZrO_2_ powder at 200 W provides the best in-melt and uniform gradient mixing with the basic material, thereby making it the most promising strategy for novel NdFeB coating preparation under laser-assisted conditions. 

### 3.2. Spectroscopic Surface Analysis

**Figure 12:**
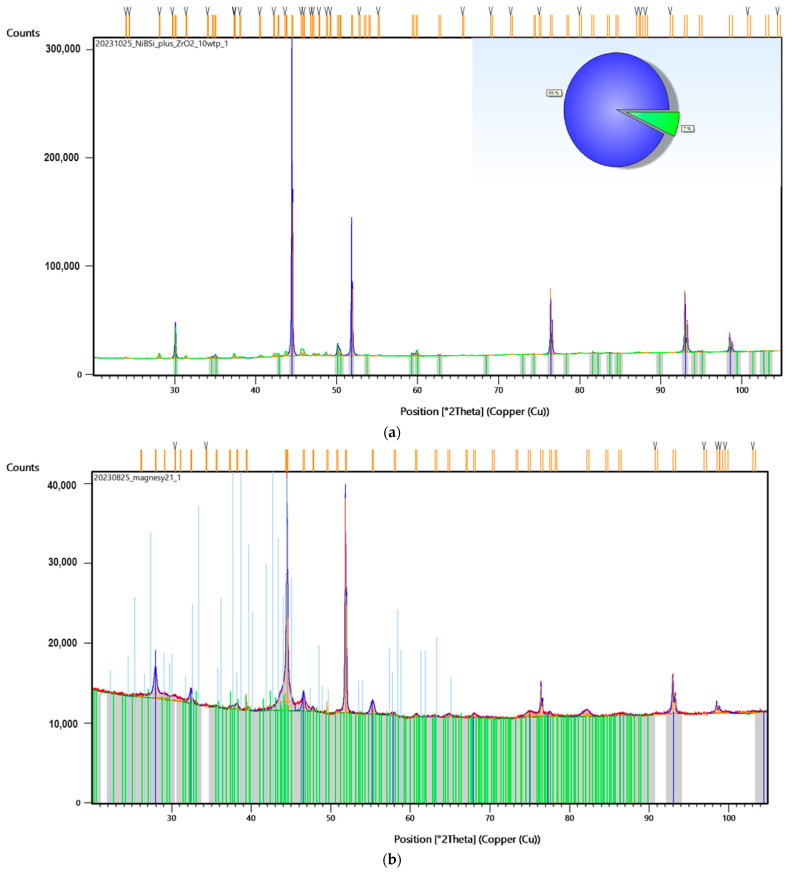
XRD analysis of: (**a**) powder used for Sample 3 coating; (**b**) Sample 3. Colorful lines refer to the peaks recognized by data base showing accuracy of their assignment to the specific elements and materials.

**Figure 13:**
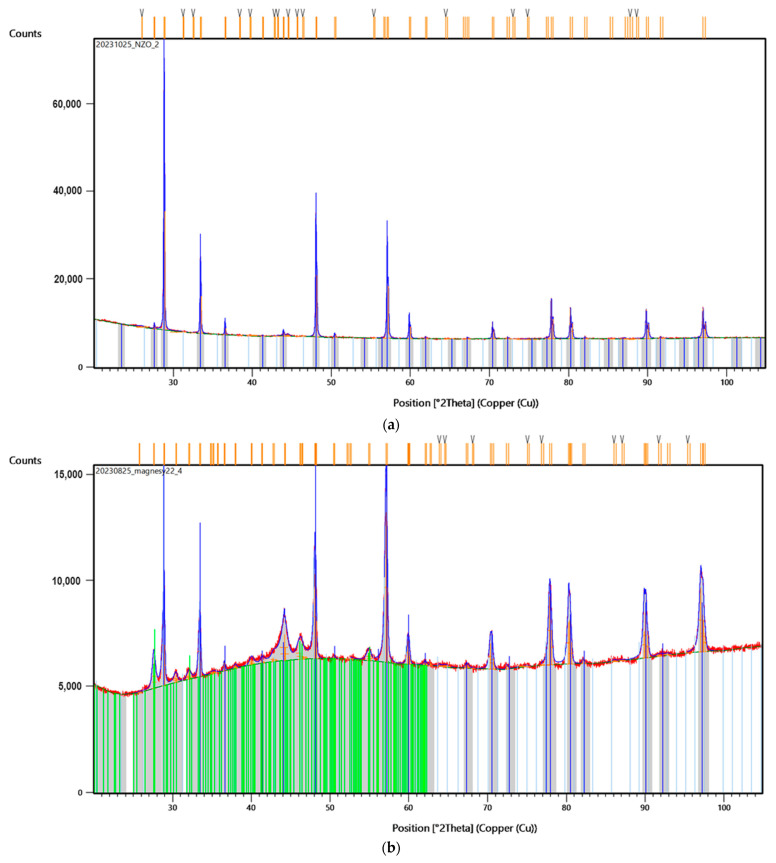
XRD of: (**a**) powder used for Sample 4 coating; (**b**) Sample 4. Colorful lines refer to the peaks recognized by data base showing accuracy of their assignment to the specific elements and materials.

To investigate the superficial changes of the samples after laser-assisted modification, XRD analysis was performed. First, the authors compared the diffractograms of the NiBSi/ZrO_2_ and Nd/ZrO_2_ powders (Figure 12a and Figure 13a, respectively). The results clearly demonstrate that laser treatment caused significant changes in the crystalline structure of both powders. Their diffractograms exhibited typical reflections at 30°, 44°, 52°, 76°, and 93°, respectively, which also appear in Sample 3’s diffractogram (Figure 12b), but with lower intensity. 

**Figure 14:**
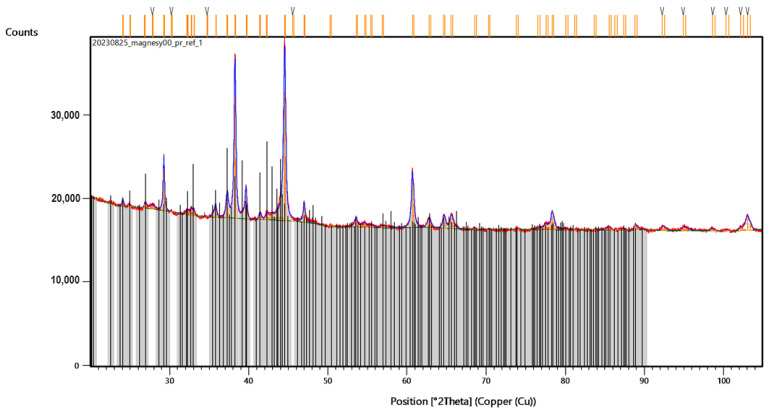
XRD analysis of NdFeB raw magnet. Colorful lines refer to the peaks recognized by data base showing accuracy of their assignment to the specific elements and materials.

Similar effects appear in the case of Nd/ZrO_2_, where the same reflections can be observed for both the raw powder and modified structure (Figure 13b). At the same time, reflections typical of NdFeB magnets (Figure 14) are missing or have a much lower intensity. This proves successful modification, via powder mixing, with the surface and new coating formation. 

**Figure 15:**
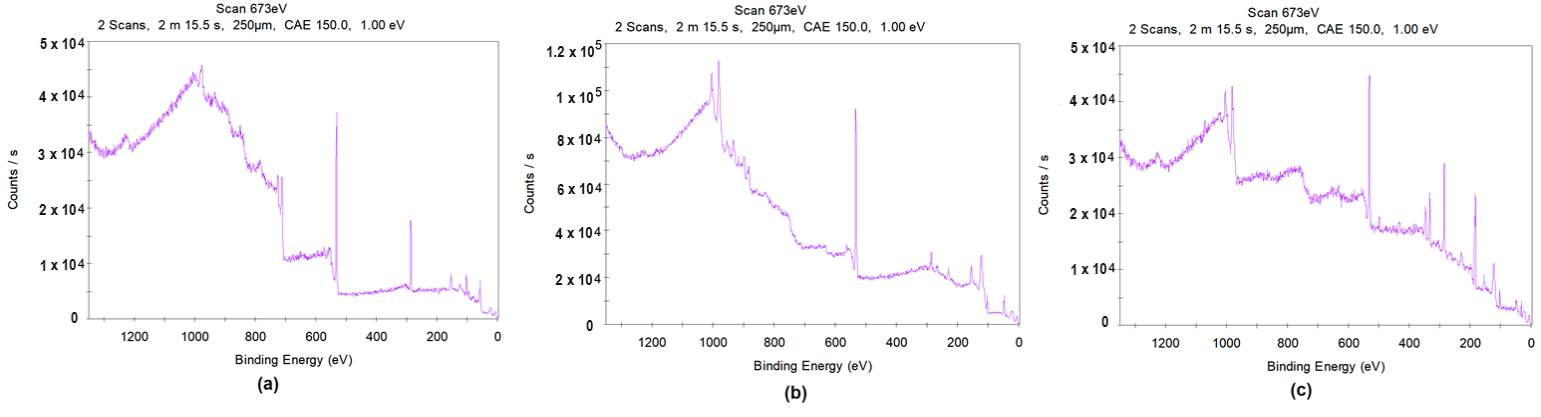
XPS analysis of the: (**a**) raw NdFeB magnet; (**b**) Sample 3; (**c**) Sample 4.

To obtain the full amount of knowledge regarding the chemical composition and states of the prepared samples, XPS analysis was conducted. The results for both the raw magnet and for Samples 3 and 4 are shown in Figure 15.

As shown in Figure 15a, the elemental composition of the unmodified magnet complies with SEM/EDS as well as XRD analysis. The spectrum reveals peaks corresponding to Nd (3d), Fe (2p), and B (1s). A peak from oxygen is also present; this is a consequence of oxidation of the uncoated sample (1s state).

Sample 3’s spectrum (Figure 15b) reveals peaks typical for Ni (2p), Nd (3d), Pr (3d), Ce (3d), Fe (2p), and O (1s); this demonstrates successful modification via laser cladding and good mixing with the substrate. No peaks corresponding to B were spotted. The XPS of Sample 4 (Figure 15c) reveals the presence of elements such as Ce (3d), Ni (2p), Nd (3d), Pr (3d), Fe (2p), and O (1s). In this case, comparing peaks intensity reveals that the elemental content of the modifiers exceeds the amount of neodymium, thereby proves successful coating. In all cases, peaks from C (1s) are also present. This is due to the presence of CO_2_ in the atmosphere, and thus carbon presence is negligible.

## 4. Conclusions

The aim of this study was to verify whether it is possible to permanently modify NdFeB magnets, which are characterized by their brittle nature, using laser cladding techniques to improve their durability and resistance through the formation of nanostructured coatings using four different micropowders. During the research, four different powders were used under various parameters in order to create superficial coatings. Cross-sectional analysis revealed that the thickness of all coatings varied between 300 and 340 µm; this decreased as the power value increased. The thickest layers were obtained for NiBSi/ZrO_2_ and Nd/ZrO_2_. In addition, no heat affecting zones could be distinguished.

Our experiments showed that by adjusting laser power and the application of the shield gas it is possible to create nanoarchitectured protecting layers on the NdFeB substrates on a large scale without causing degradation and cracking. After each test, the samples were investigated for their structure, morphology, roughness, and chemical composition. Their crystalline structure was also evaluated using the XRD method. Successful laser cladding resulting in powder mixing with the substrate and coating formation was also confirmed using the XPS method. Our results clearly demonstrate that the presence of oxygen in chemical composition of powders applied during laser-assisted modification significantly affects the process and improves the materials’ melting, mixing, and nanostructuring. These results indicate that modification of brittle materials with a fragile nature exclude the use of high power values during laser cladding. This can be performed by using metal oxides, as metallic powders without an additional matrix are not sufficient to cover the surface and undergo satisfactory in-melt. Our EDS elemental analyses revealed that laser cladding for Samples 3 and 4 led to better mixing with the basic material and that the powder was more evenly distributed at the surface when compared to spongy Ti and ZrO_2_. The best results were obtained for the NiBSi/ZrO_2_ micropowder.

Our flux density studies, obtained from both the field analysis and laboratory tests, showed that the magnets’ parameters differ from the datasheet according to the sample size and coating selection. Further modification of the surface also changes the flux density value. After the coating process, the magnets must be re-magnetized.

Our future research will focus on long-term stability studies of newly developed coatings and their impact on life cycle and wear resistance improvement. 

## Figures and Tables

**Table 1:** Coatings thickness.

Sample	Coating Thickness, µm
Ti/100 W	380
Ti/150 W	313
Ti/175 W	297
Ti/200 W	288
ZrO_2_/100 W	342
ZrO_2_/150 W	335
ZrO_2_/175 W	326
ZrO_2_/200 W	312
NiBSi/ZrO_2_/200 W	1351
Nd/ZrO_2_/200 W	337

## Data Availability

Data available on request.

## Supplementary Materials

The following supporting information can be downloaded at: https://www.mdpi.com/article/10.3390/nano14050431/s1, Figure S1. Magnetic flux density according to the distance from the magnet’s middle for size of 20 × 10 × 5 mm, FEM analysis using ANSYS Maxwell software. Figure S2. Laboratory tests of magnetic flux density as a function of temperature: green line: no-coating; red line: commercial Cu-Ni-Cu magnet coating. Figure S3. SEM images of the powders used for coatings preparation: (a) Ti; (b) ZrO_2_ stabilized with Y; (c) NiBSi/ZrO_2_; (d) Nd/ZrO_2_. Figure S4. EDS analysis of the powders used for Samples 1 and 2: (a) Ti; (b) ZrO_2_ stabilized with Y. Figure S5. EDS analysis of the uncoated NdFeB magnet. Figure S6. Surface roughness analysis of the uncoated NdFeB magnet. Figure S7. EDS analysis of Sample 1: (a) 100 W; (b) 150 W; (c) 175 W; (d) 200 W. Figure S8. Surface roughness analysis of Sample 1: (a) 100 W; (b) 150 W; (c) 175 W; (d) 200 W. Figure S9. EDS analysis of Sample 2: (a) 100 W; (b) 150 W; (c) 175 W; (d) 200 W. Figure S10. Surface roughness analysis of Sample 2: (a) 100 W; (b) 150 W; (c) 175 W; (d) 200 W. Figure S11. Surface roughness analysis of Sample 3. Figure S12. Surface roughness analysis of Sample 4.
