# Supplementary material for: Commercial-Scale Modification of NdFeB Magnets under Laser-Assisted Conditions
Source: Nanomaterials (Basel). 2024 Feb 27;14(5):431. doi: 10.3390/nano14050431 (PMC10934298; doi:10.3390/nano14050431)
Supplement: Supplementary file 1 [file nanomaterials-14-00431-s001.zip › nanomaterials-2861446-supplementary.pdf]

Supplementary figures

Commercial-scale modification of NdFeB magnets under laser-assisted conditions

I Magnetic flux density

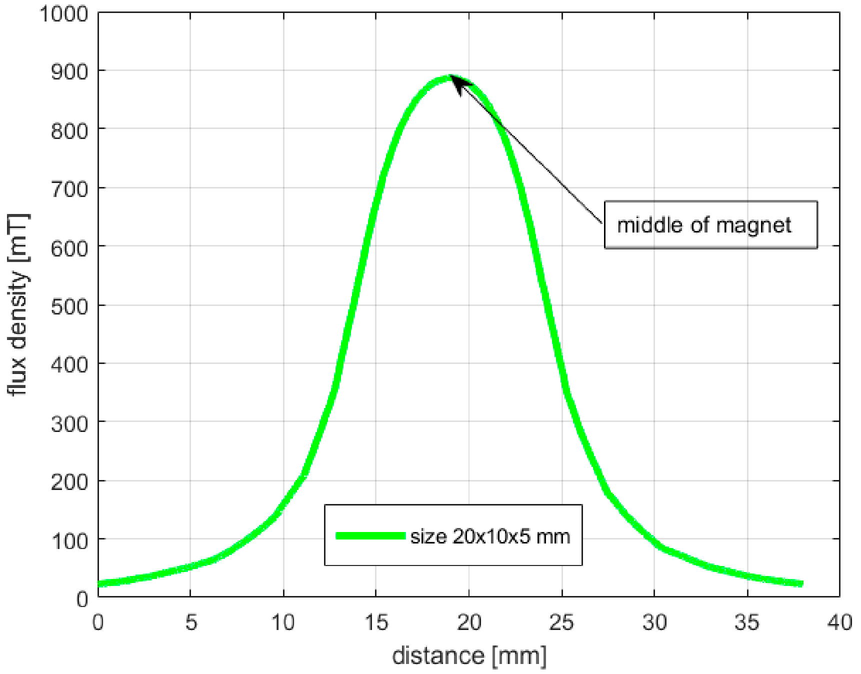

Figure S1. Magnetic flux density according to the distance from the magnet's middle for size of 20x10x5 mm, FEM analysis using ANSYS Maxwell software.

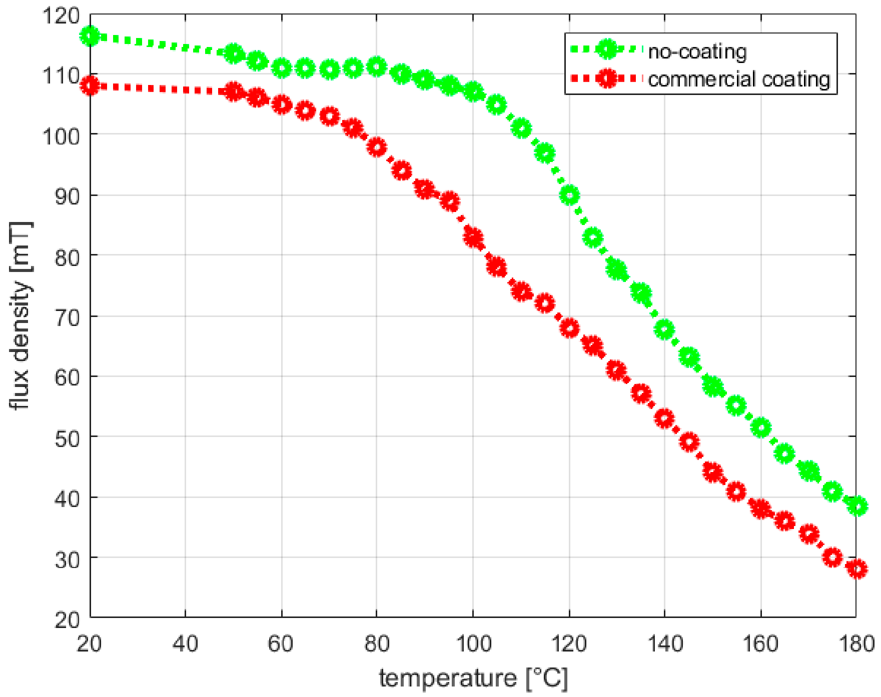

Figure S2. Laboratory tests of magnetic flux density as a function of temperature: green line: no-coating; red line: commercial Cu-Ni-Cu magnet coating.

II Morphology study

- ***Powders used for coatings preparation: spongy Ti, ZrO_2 stabilized with Y and self-made powder mixes: NiBSi/ZrO_2 and Nd/ZrO_2***

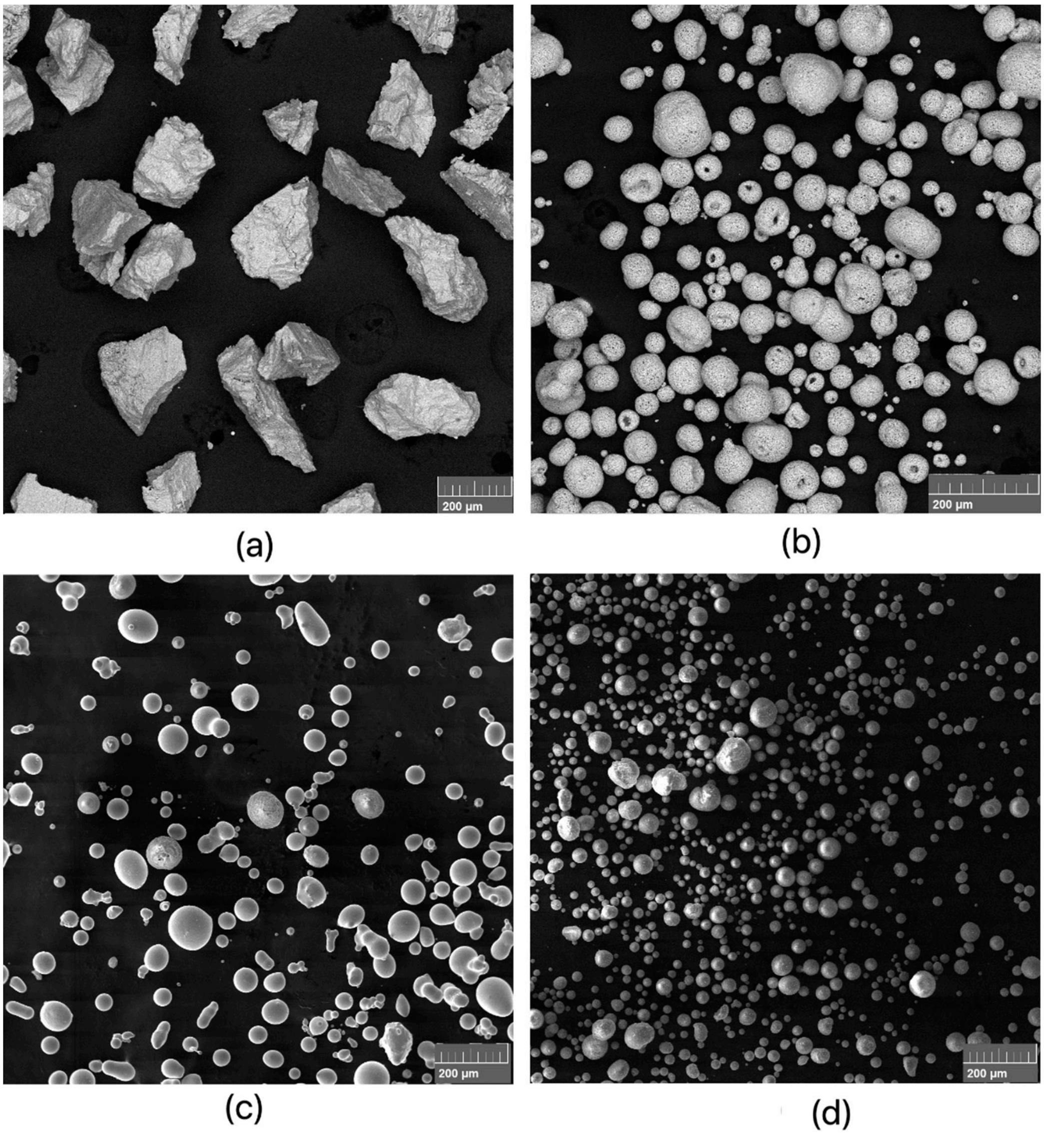

Figure S3. SEM images of the powders used for coatings preparation: (a) Ti; (b) ZrO_2 stabilized with Y; (c) NiBSi/ZrO_2; (d) Nd/ZrO_2.

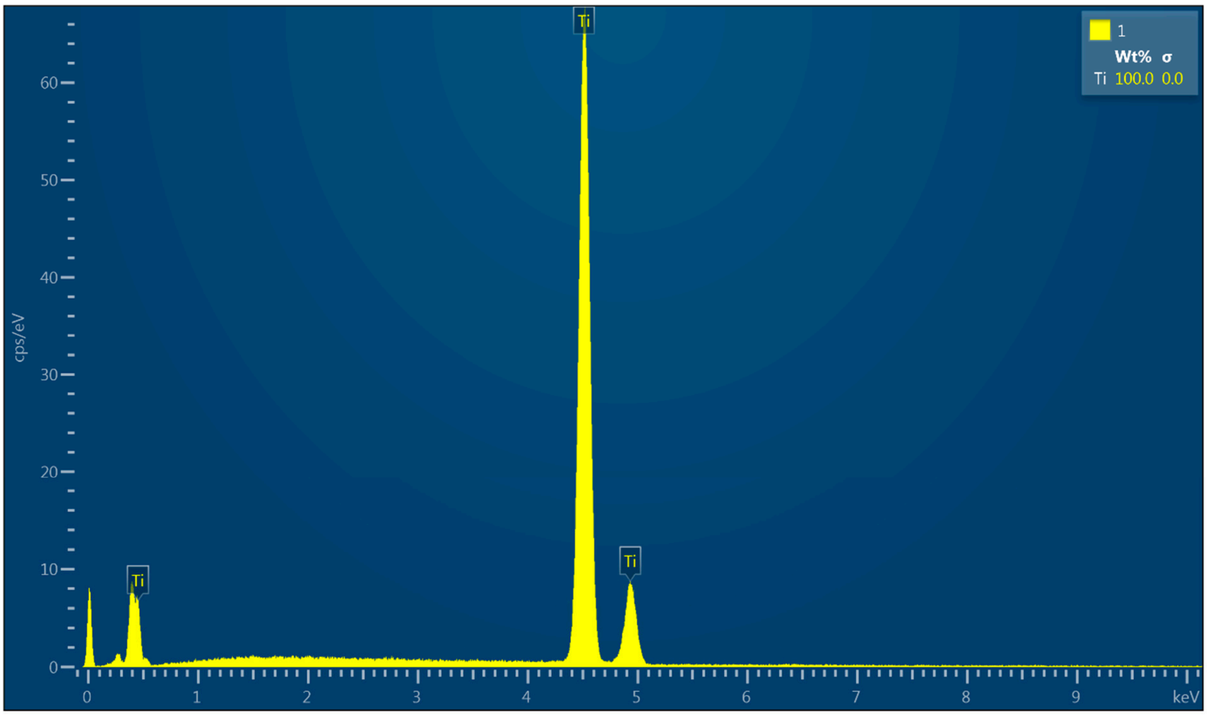

(a)

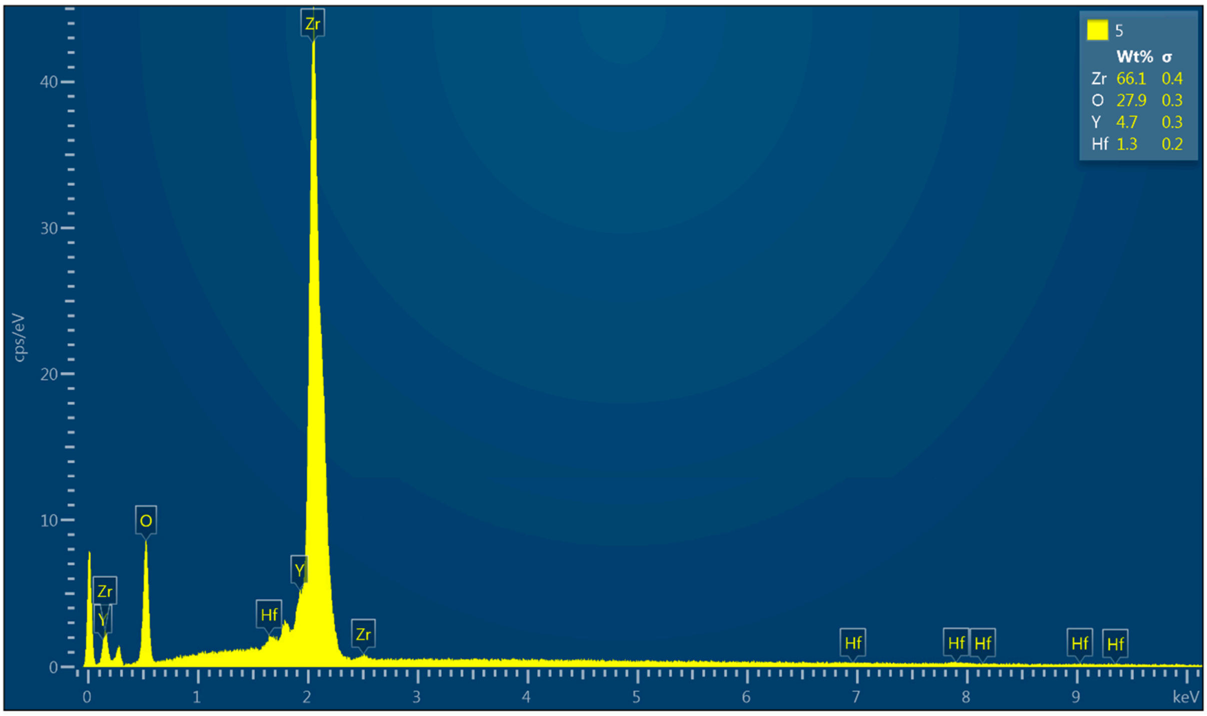

(b)

Figure S4. EDS analysis of the powders used for Samples 1 and 2: (a) Ti; (b) ZrO_2 stabilized with Y.

- ***Raw NdFeB magnet***

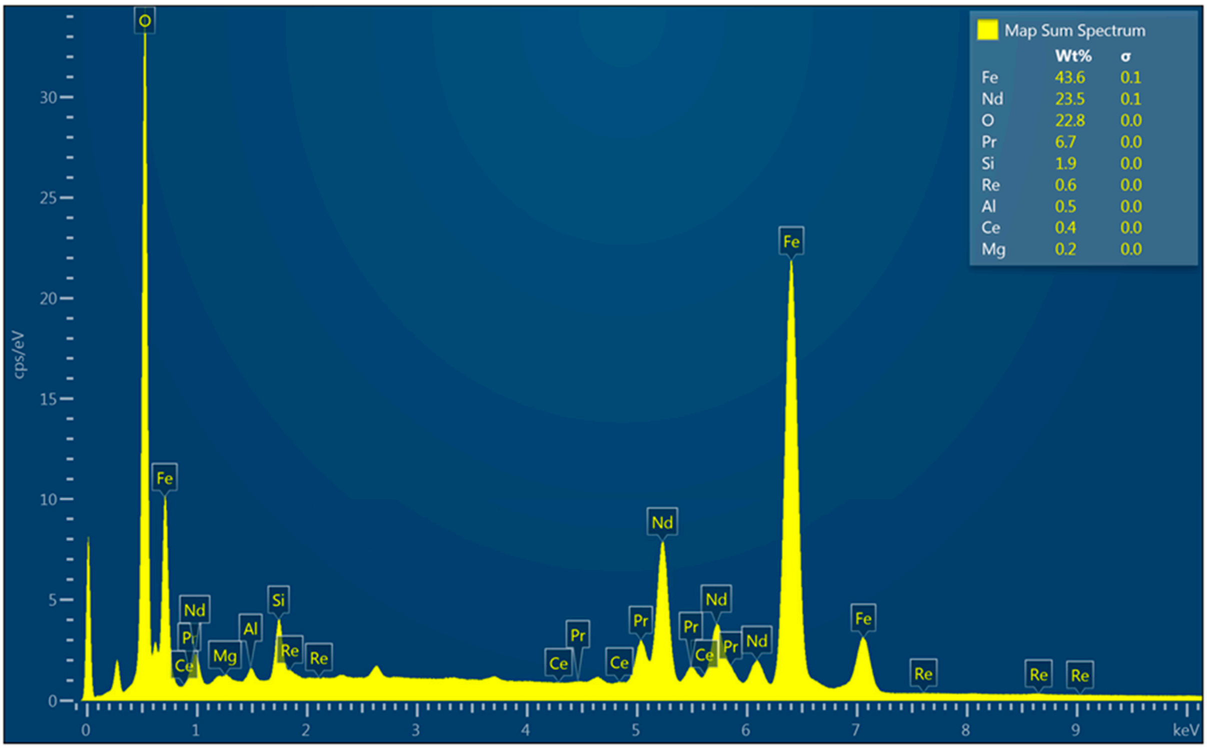

Figure S5. EDS analysis of the uncoated NdFeB magnet.

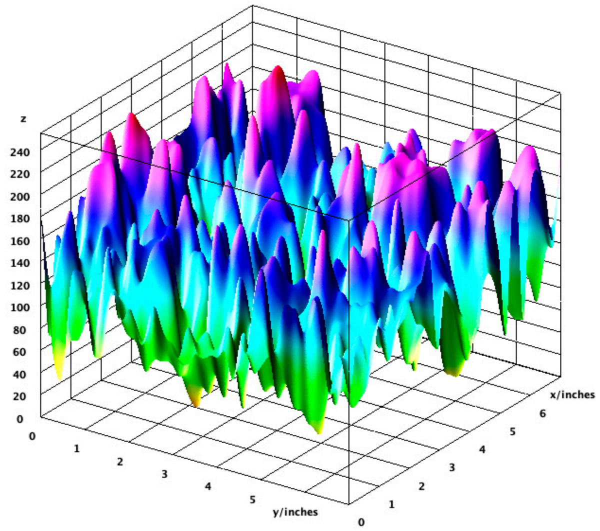

Figure S6. Surface roughness analysis of the uncoated NdFeB magnet.

- *Sample 1 – coating from spongy Ti, applied power of 100 W, 150 W, 175 W and 200 W.*

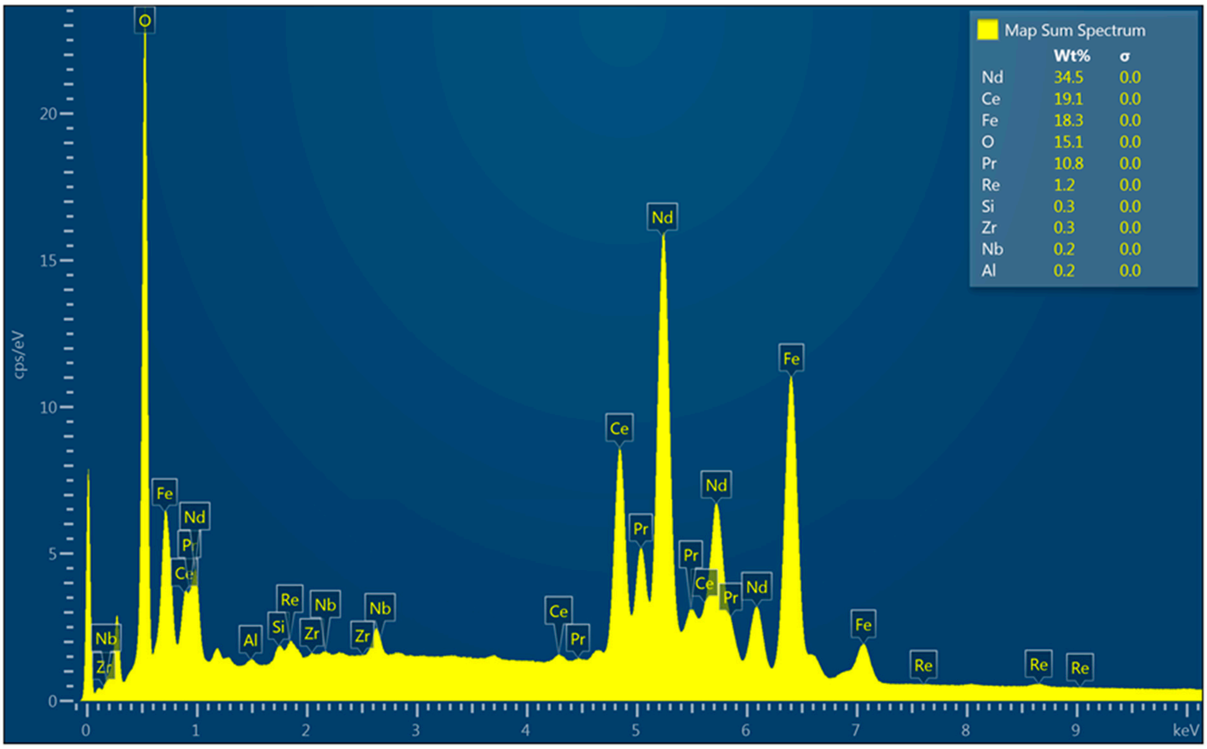

(a)

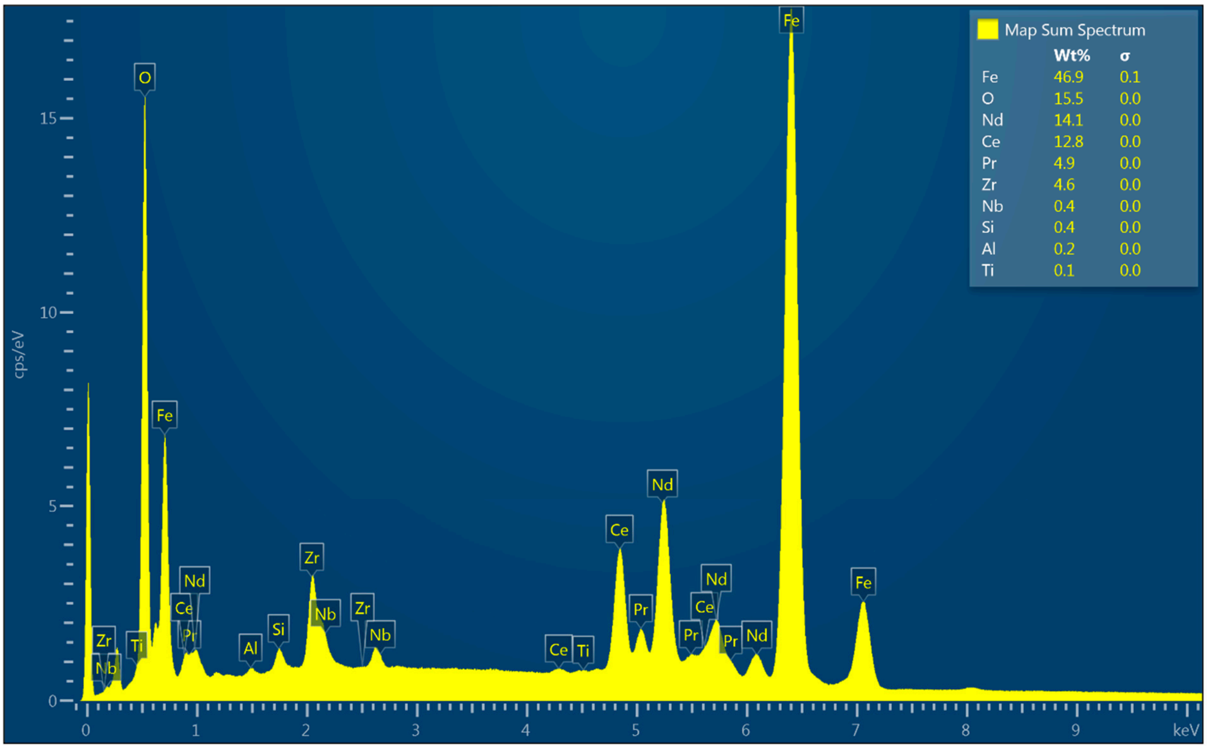

(b)

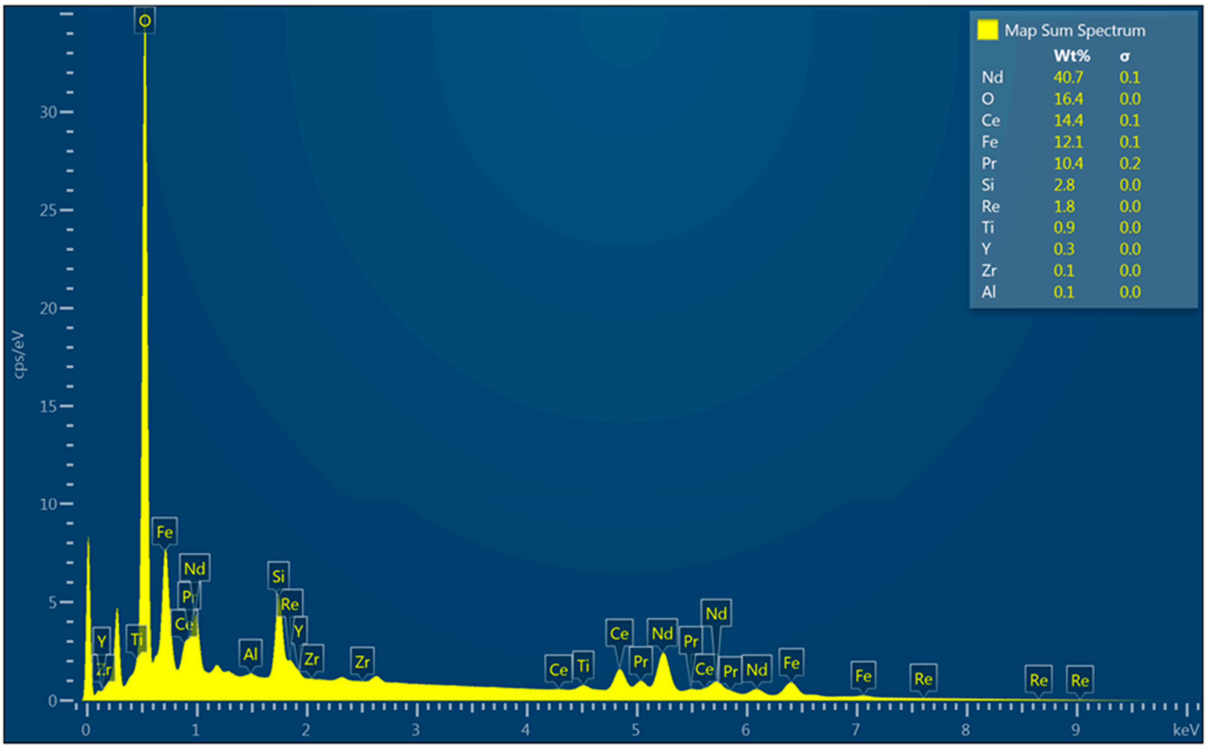

(c)

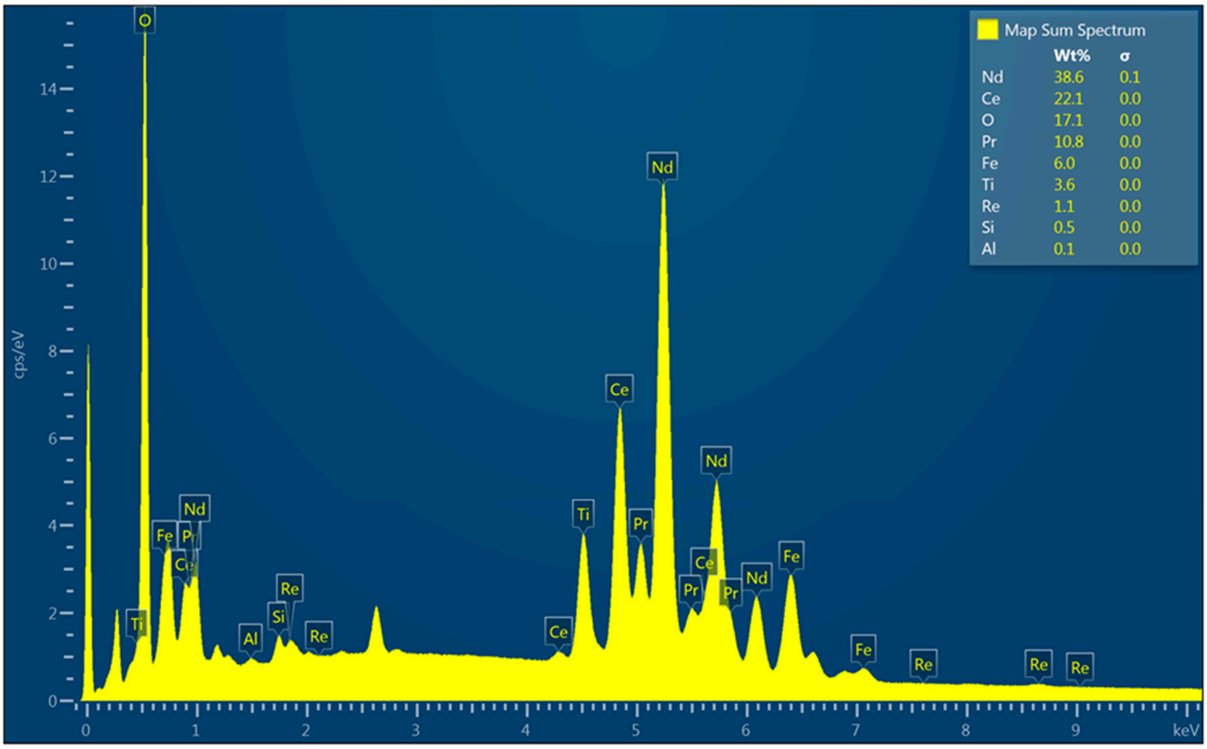

(d)

Figure S7. EDS analysis of Sample 1: (a) 100 W; (b) 150 W; (c) 175 W; (d) 200 W.

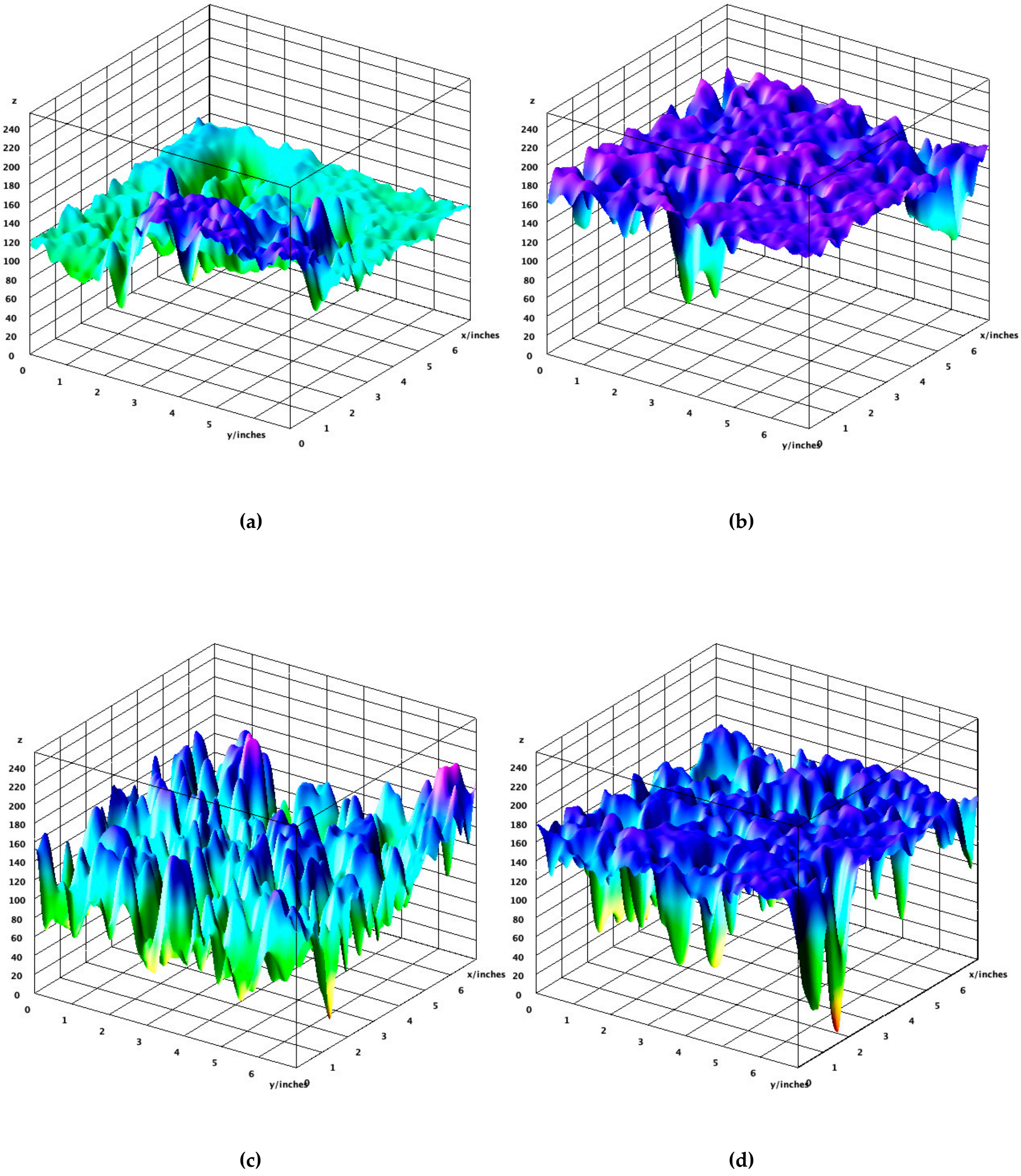

Figure S8. Surface roughness analysis of Sample 1: (a) 100 W; (b) 150 W; (c) 175 W; (d) 200 W.

- *Sample 2 – coating from ZrO_2 stabilized with Y, applied power of 100 W, 150 W, 175 W and 200 W*

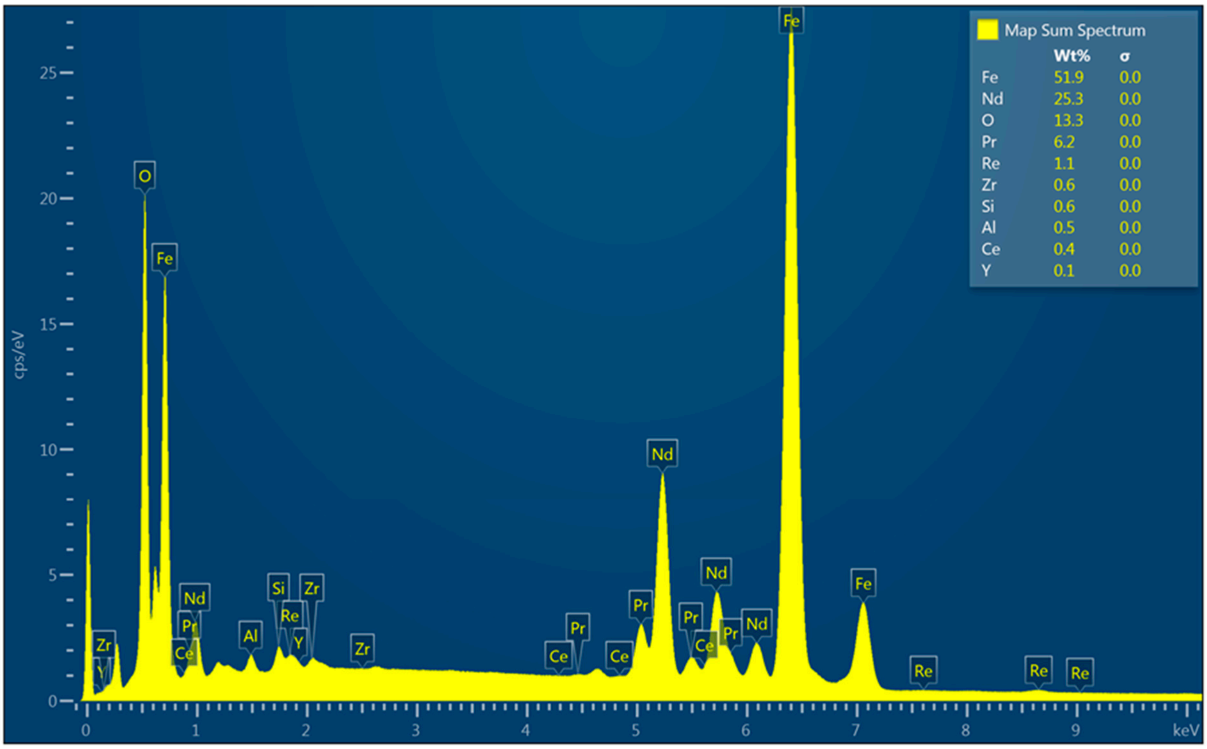

(a)

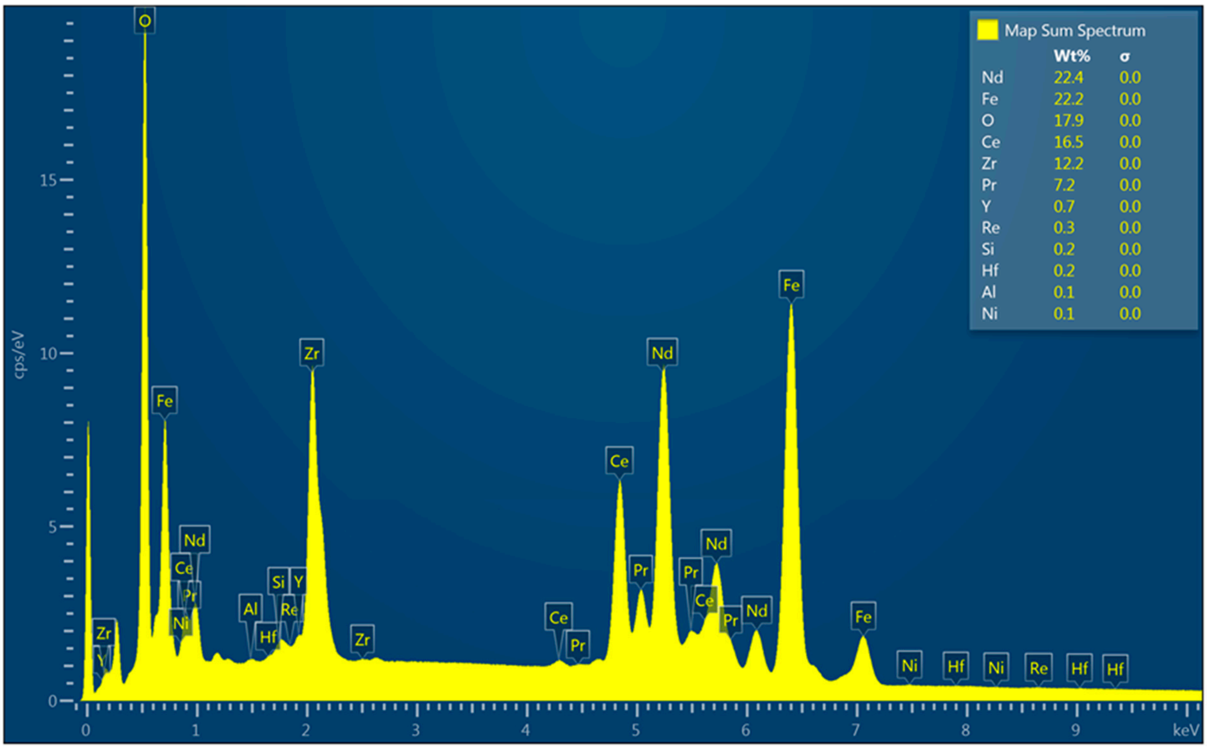

(b)

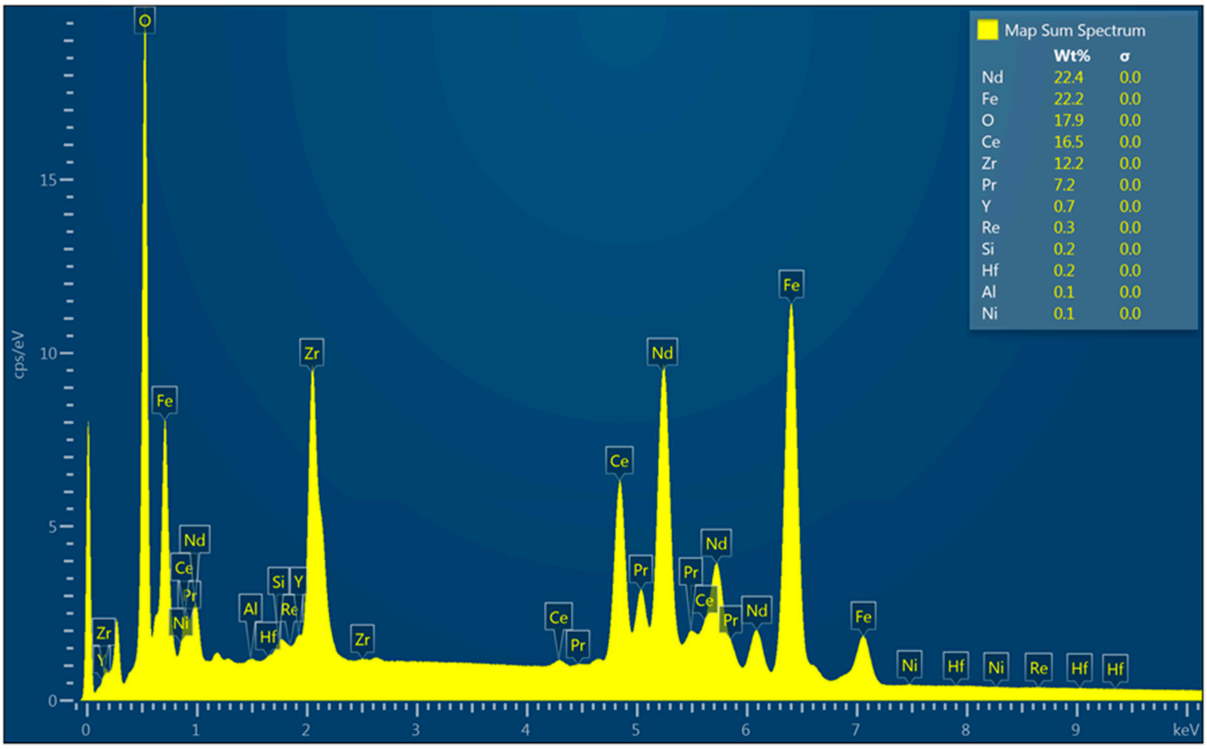

(c)

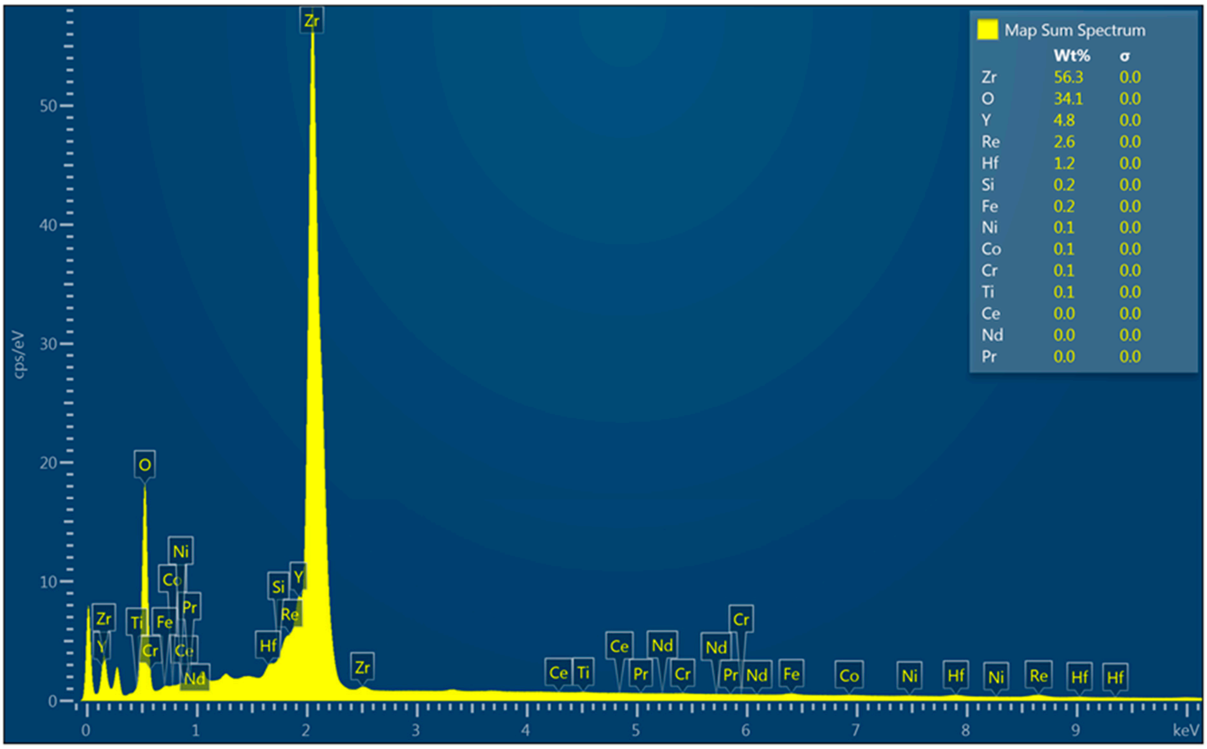

(d)

Figure S9. EDS analysis of Sample 2: (a) 100 W; (b) 150 W; (c) 175 W; (d) 200 W.

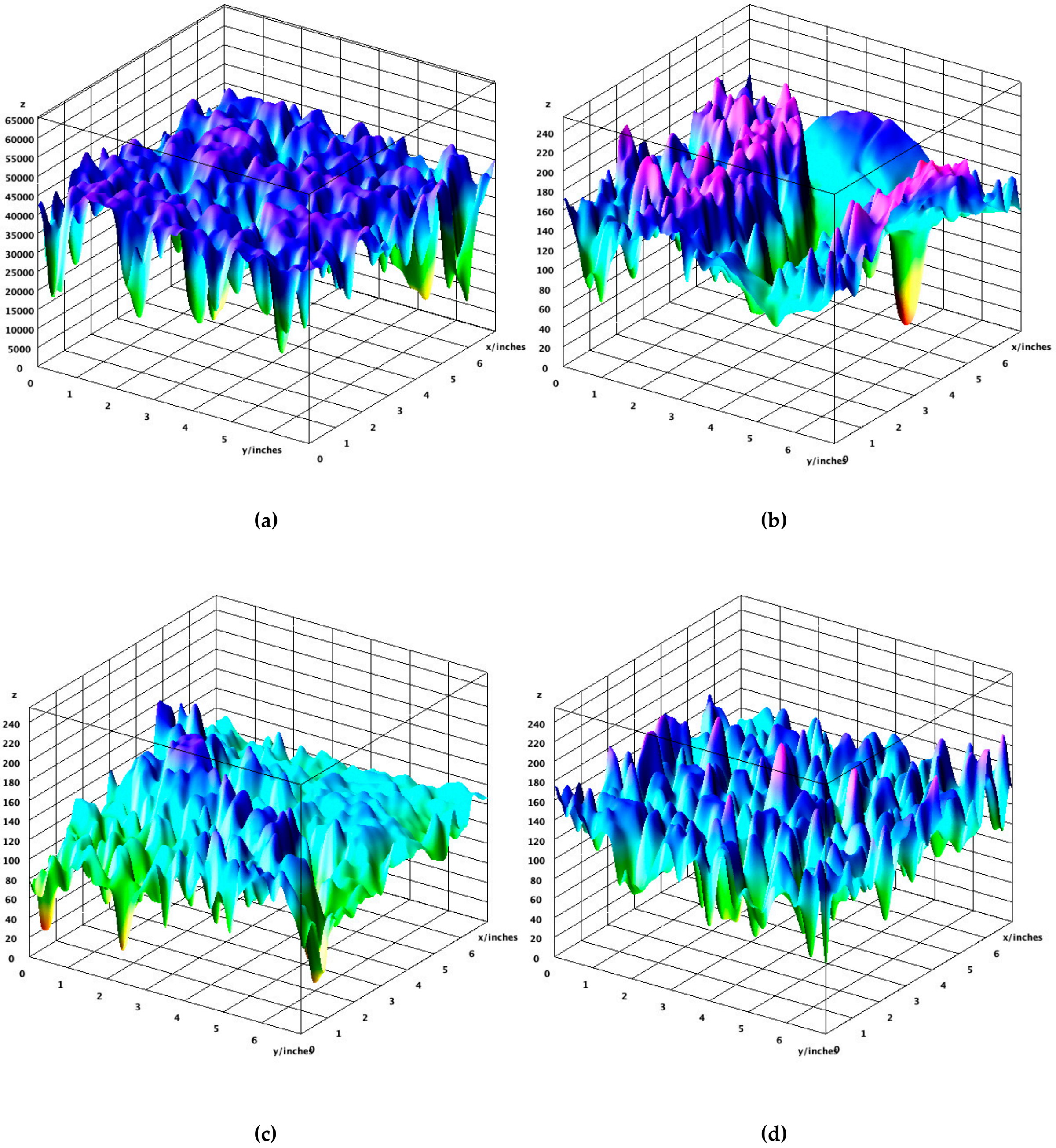

Figure S10. Surface roughness analysis of Sample 2: (a) 100 W; (b) 150 W; (c) 175 W; (d) 200 W.

- ***Sample 3 - coating from self-made powder mix NiBSi/ZrO_2, applied power of 200 W***

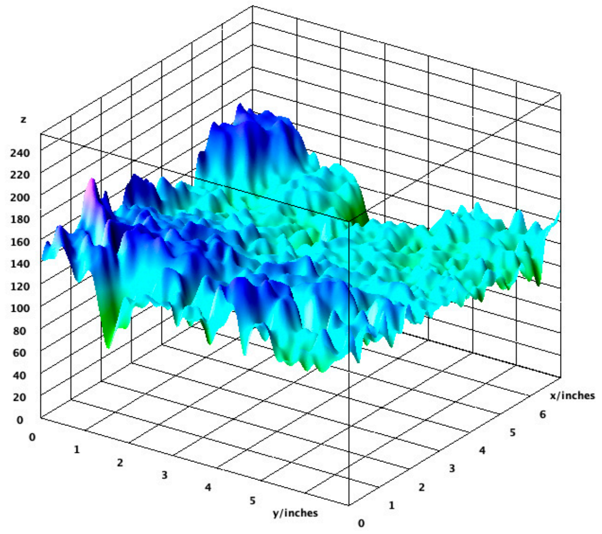

Figure S11. Surface roughness analysis of Sample 3

- ***Sample 4 – coating from self-made powder mix Nd/ZrO_2, applied power of 200 W***

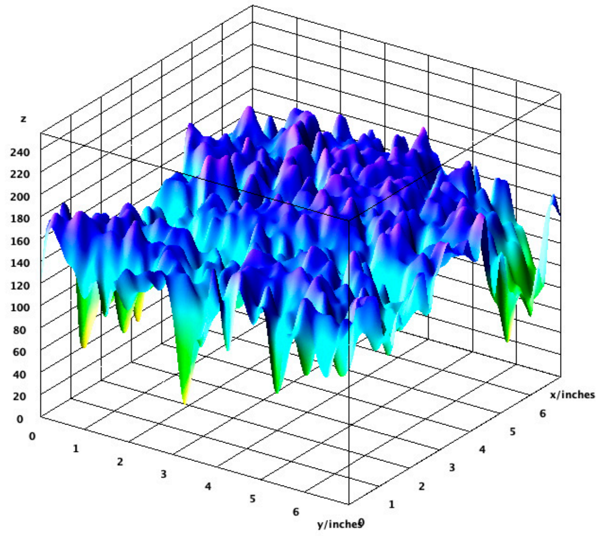

Figure S12. Surface roughness analysis of Sample 4.
